# Glycerol Affects Root Development through Regulation of Multiple Pathways in Arabidopsis

**DOI:** 10.1371/journal.pone.0086269

**Published:** 2014-01-22

**Authors:** Jun Hu, Yonghong Zhang, Jinfang Wang, Yongming Zhou

**Affiliations:** 1 National Key Laboratory of Crop Genetic Improvement, Huazhong Agricultural University, Wuhan, China; 2 College of Plant Science and Technology, Huazhong Agricultural University, Wuhan, China; National Taiwan University, Taiwan

## Abstract

Glycerol metabolism has been well studied biochemically. However, the means by which glycerol functions in plant development is not well understood. This study aimed to investigate the mechanism underlying the effects of glycerol on root development in *Arabidopsis thaliana*. Exogenous glycerol inhibited primary root growth and altered lateral root development in wild-type plants. These phenotypes appeared concurrently with increased endogenous glycerol-3-phosphate (G3P) and H_2_O_2_ contents in seedlings, and decreased phosphate levels in roots. Upon glycerol treatment, G3P level and root development did not change in glycerol kinase mutant *gli1*, but G3P level increased in *gpdhc1* and *fad-gpdh* mutants, which resulted in more severely impaired root development. Overexpression of the *FAD-GPDH* gene attenuated the alterations in G3P, phosphate and H_2_O_2_ levels, leading to increased tolerance to exogenous glycerol, which suggested that FAD-GPDH plays an important role in modulating this response. Free indole-3-acetic acid (IAA) content increased by 46%, and *DR5pro::GUS* staining increased in the stele cells of the root meristem under glycerol treatment, suggesting that glycerol likely alters normal auxin distribution. Decreases in *PIN1* and *PIN7* expression, *β*-glucuronidase (GUS) staining in plants expressing *PIN7pro::GUS* and green fluorescent protein (GFP) fluorescence in plants expressing *PIN7pro::PIN7-GFP* were observed, indicating that polar auxin transport in the root was downregulated under glycerol treatment. Analyses with auxin-related mutants showed that TIR1 and ARF7 were involved in regulating root growth under glycerol treatment. Glycerol-treated plants showed significant reductions in root meristem size and cell number as revealed by *CYCB1;1pro::GUS* staining. Furthermore, the expression of *CDKA* and *CYCB1* decreased significantly in treated plants compared with control plants, implying possible alterations in cell cycle progression. Our data demonstrated that glycerol treatment altered endogenous levels of G3P, phosphate and ROS, affected auxin distribution and cell division in the root meristem, and eventually resulted in modifications of root development.

## Introduction

Terrestrial plants have evolved effective and intricate mechanisms to survive biotic and abiotic stress in soil. One good example of such a mechanism is the plasticity of plant root development. Root development involves cell division and elongation at the root meristem, lateral root primordium (LRP) initiation and lateral root (LR) formation. It has been well documented that hormones, such as auxin, are involved in this highly complex and dynamic process [1].

Asymmetric auxin distribution, which involves dynamic changes in the auxin gradient [2], play a crucial role in root development. Maintaining the correct auxin gradient is necessary for major root developmental events, such as apical-basal axis formation and LR development [3]–[5]. Asymmetric auxin distribution can be modulated by intercellular polar auxin transport, which is a specialized delivery system whereby plants transport indole-3-acetic acid (IAA) from auxin sources in the shoot to sink tissues such as roots. Polar auxin transport depends on the directional cellular localization of auxin transport components, such as members of the auxin efflux carrier PIN-FROMER (PIN) protein family [6], the auxin influx carrier AUX1/LIKE-AUXIN (AUX1/LAX) family [7], and the of ATP-dependent multi-drug resistance/P-glycoprotein (MDR/PGP)-type ABC transporters family [8]. Environmental and/or genetic interference with auxin transport can alter root meristem activity, thus affecting root development [6], [9], [10].

LRs originate exclusively from the pericycle cell layer, where the LRP is initiated and emerges from the primary root (PR) [11]. Exogenous application or endogenous overproduction of auxin induces LR initiation [12]. Mutations in genes involved in auxin homeostasis, signaling and transport cause defects in the development of LRs [5]. TIR1 encodes an auxin receptor that interacts with Aux/IAA transcriptional repressor proteins, such as SLR/IAA14, and mediates their degradation [13]–[15]. The degradation of Aux/IAA repressors allow ARFs, a large class of transcriptional regulators involved in plant growth responses to auxin (such as ARF7 and ARF19), to activate the transcription of auxin-responsive genes [16].

In addition to plant hormones, other factors such as reactive oxygen species (ROS) and nutrients are also important in root development. ROS acts as important second messengers in the perception of stresses [17]–[19]. ROS produced by NADPH oxidase/RHD2 regulate plant root cell elongation [20]. Recent studies have shown that ROS homeostasis at least partially regulates root cell proliferation and elongation at the transcriptional level [21]. The levels of nutrients such as nitrogen and phosphate also affect root development [22]–[26], for example, low phosphate availability alters lateral root development in Arabidopsis by modulating auxin sensitivity [19].

Glycerol is a common metabolite. In microorganisms and invertebrates, glycerol protects against stress, especially anaerobic and osmotic stresses [27]–[29]. Although high level of glycerol have been found in a few species, such as *Candida glycerolgenesis* and *Dunaliella parva* [30], [31], only trace amounts can be detected in higher plants [32]. Exogenous glycerol can have dramatic effects on plant growth [33]–[35]. For example, the application of glycerol to barley and spinach leaves affects photosynthetic carbon assimilation [34]. The addition of 50 mM glycerol to medium without sucrose imposes sequential physiological and biochemical effects on sycamore cells, such as a rapid accumulation of glycerol-3-phosphate (G3P) at the expense of cytoplasmic phosphate (Pi), inhibition of glucose-6-phosphate isomerase activity and prevention of triose phosphate recycling back to hexose phosphate, which resulted in the arrest of cytosolic and plastidial pentose phosphate pathways [35]. Supplying glycerol stimulated triacylglycerol synthesis in developing *Brassica napus* seeds [36]. The application of 50 mM glycerol resulted in reduced oleic acid content, increased salicylic acid content and increased Pathogenesis-related (*PR-1*) gene expression in wild-type Arabidopsis [37], [38]. Glycerol affects cytoskeletal rearrangements during the induction of somatic embryogenesis [39] and represses the catabolism of the major phospholipid phosphatidylcholine while facilitating its synthesis [40]. The information above suggests that glycerol may exert several different effects through various pathways.

Glycerol is a precursor of G3P, which is a key metabolite that carries reducing equivalents from the cytosol to the mitochondria for oxidative phosphorylation and acts as the backbone of glycerolipids. Glycerol and G3P metabolism involves several key enzymes: glycerol kinase, mitochondrial FAD-G3P dehydrogenase (FAD-GPDH) and NAD^+^-dependent G3P dehydrogenase (GPDH) presented in both cytosol and chloroplasts [29], [41]–[43]. Specifically, glycerol kinase phosphorylates glycerol to G3P and consumes ATP simultaneously. G3P is oxidized to dihydroxyl-acetone phosphate (DHAP) by FAD-GPDH, which converts FAD to FADH_2_. Both of GPDH isoforms regenerates G3P by consuming DHAP and NADH. The *Arabidopsis* mutant *sdp6* overaccumulates G3P and exhibits seedling developmental arrest after germination [44]. Disruption of the *GPDHc1* gene in the *gpdhc1* mutant results in impaired glycerol metabolism and increased cellular ROS [45]. The manipulation of G3P content has been explored as a mechanism for regulating plant metabolism. Heterologous expression of glycerol metabolism-related genes from yeast and *Escherichia coli* has been found to increase G3P and lipid content in *Brassica napus* seeds and to alter glycerolipid flux in Arabidopsis leaves, respectively [46], [47], but overexpression of endogenous native GPDH isoforms does not alter basal G3P or fatty acid levels in Arabidopsis [42], [43]. It has been reported that G3P level is important for basal resistance to the fungus *Colletotrichum higginsianum* in Arabidopsis [42] and that G3P serves as an inducer of systemic acquired resistance at a very early time point [43]. G3P contributes to systemic acquired resistance against stripe rust in wheat [48]. Very recently, it was showed that normal G3P pool is required for stability of defense proteins [49]. G3P is also relevant to mitochondrial respiration through G3P shuttle and plays an important role in modulating the NADH/NAD^+^ ratio and cellular redox state in plant cells [45]. However, it is unclear how these factors affect root system growth from a cellular and developmental perspective.

This study aimed to investigate the mechanisms underlying the effects of glycerol on root development in Arabidopsis. Mutant analysis combined with biochemical assays demonstrated that the alterations of G3P and ROS levels in seedlings and phosphate level in roots are associated with root growth modifications under glycerol treatment. The effects on root architecture were further linked to altered auxin transport and distribution using auxin signaling-related mutants and to decreased cell cycle progression in the root meristem.

## Results

### Exogenous Glycerol Inhibits Primary Root Length and Alters Lateral Root Primordium Development

**Figure 1:**
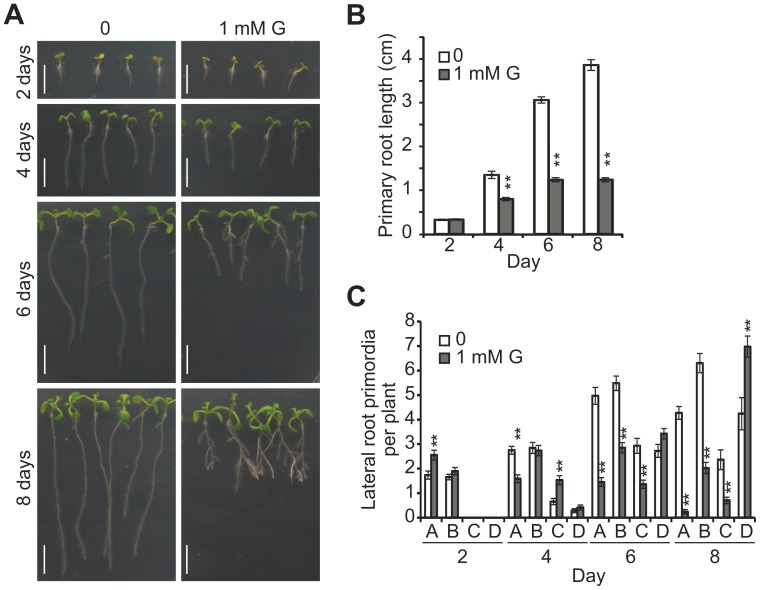
Exogenous glycerol inhibits Arabidopsis primary root growth and has variable effects on lateral root primordia. Wild-type seedlings were grown on plates containing 0.5×Murashige and Skoog (MS) medium with 1 mM glycerol for the indicated number of days. (A) Root development of seedlings grown on control (left) and 1 mM glycerol media (right) at 2–8 days post germination (dpg). Bar = 0.5 cm. (B) Quantification of the primary root (PR) lengths of the seedlings grown under the conditions described in (A). (C) Quantification of the lateral root primordia (LRPs) of the seedlings grown under the conditions described in (A). The developmental stage of each LRP was classified according to Zhang *et al.* (1999): Stage A, up to three cell layers; Stage B, unemerged LR, but more than three cell layers; Stage C, emerged LR <0.5 mm in length; Stage D, LR longer than 0.5 mm. The data are presented as the mean ± SE (n = 10). Asterisks indicate a significant difference at p<0.05 (*) or p<0.01 (**) by Student’s t-test.

When wild-type Arabidopsis plants were grown on 0.5×Murashige and Skoog (MS) media containing various concentrations of glycerol ranging from 0 to 20 mM, the primary root (PR) length of the plants grown on media containing less than 100 µM glycerol was not significantly different from the root length on the control medium (without glycerol); however, the PR length of plants grown on medium containing 1 mM glycerol was significantly shorter than the root length on the control medium (Figure S1). The inhibitory effect of glycerol on PR length was observable at 4 days post-germination (dpg) (Figure 1A). Furthermore, the inhibition became more significant with longer treatment times (Figure 1B).

The effect of glycerol treatment on the number of lateral root primordium (LRP) was also examined at the same time points used for PR length observations, following the four developmental stages proposed by Zhang *et al.* [50]: Stage A includes primordia of up to three cell layers; Stage B includes unemerged LRs that have more than three cell layers; Stage C includes emerged LRs <0.5 mm in length; and Stage D includes LRs longer than 0.5 mm. In the present study, the number of LRP at Stage A per plant under glycerol treatment was significantly higher than that under the control treatment at 2 dpg; however, this trend was reversed at 4–8 dpg (Figure 1C). The numbers of LRP at Stage B were similar between the treatment and control groups at 2–4 dpg, although the number of LRP in the treatment group was significantly reduced compared with that of the controls at 6–8 dpg (Figure 1C). The number of LRP at Stage C per plant in the treatment group was significantly increased compared with the control at 4 dpg but decreased at 6 and 8 dpg. The number of LRP at Stage D was similar between the control and the treatment groups at 2–4 dpg; however, glycerol-treated plants had significantly more LRP than the control plants at 6–8 dpg (Figure 1C). When the glycerol treatment was extended, the inhibition of PR growth was accompanied by an alteration in LRP development. The number of second-order LRP under glycerol treatment was increased compared with the control (Table S1). Furthermore, exogenous glycerol inhibited PR growth and modified LR development under dark conditions (Figure S2), suggesting that the effect of glycerol treatment on root development was not affected by light.

### Glycerol Metabolism-related Genes are Involved in Modulating Root Growth in Response to Exogenous Glycerol

**Figure 2:**
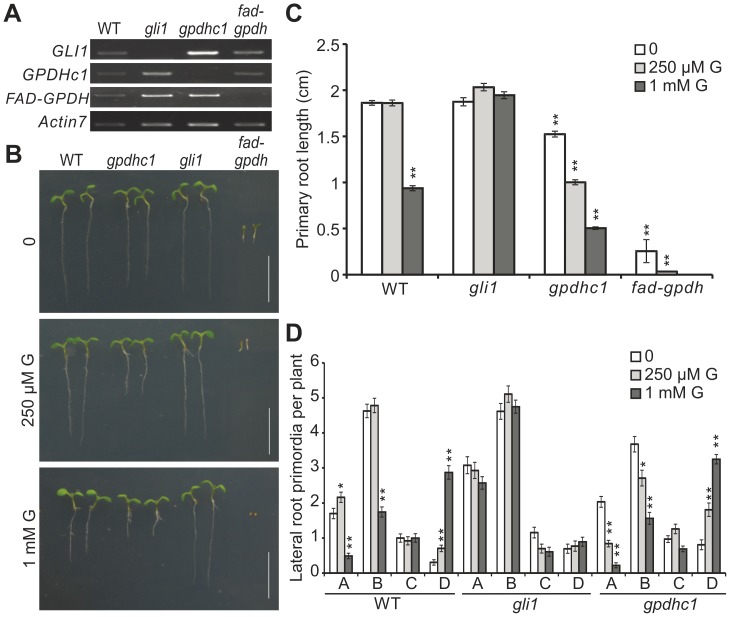
Responses of mutants with glycerol metabolism defects to exogenous glycerol. (A) Detection of *GLI1*, *GPDHc1* and *FAD-GPDH* transcripts by RT-PCR in wild-type, *gli1*, *gpdhc1* and *fad-gpdh* seedlings. Total RNA was prepared from 3-day-old seedlings. *Actin07* was used as an internal control. Wild-type and *gli1, gpdhc1* and *fad-gpdh* mutant seedlings were grown for 6 days on vertically oriented agar plates. (B) Seedlings growing on media containing various concentrations of glycerol (0, 250 µM and 1 mM) are shown. Bar = 1 cm. (C) The primary root (PR) lengths of WT and *gli1*, *gpdhc1* and *fad-gpdh* mutant seedlings were recorded. (D) Data on the number of lateral root primordia (LRP) per plant were statistically analyzed. The data are presented as the mean of 30–40 seedlings ± SE. Asterisks indicate significant differences between the treatment (250 µM and 1 mM glycerol) and control (0) (*, p<0.05; **, p<0.01) by Student’s t-test.

To test the hypothesis that the inhibition of root growth in glycerol-containing media was caused by modified glycerol catabolism in plants, we examined the root growth of several mutants defective in glycerol catabolism (*gli1*, *gpdhc1* and *fad-gpdh*) grown on media containing 0 µM, 250 µM or 1 mM glycerol. Reverse transcription polymerase chain reaction (RT-PCR) showed that the expression of the corresponding genes in these mutants was disrupted (Figure 2A). *GLI1* encodes a glycerol kinase that catalyzes the conversion of glycerol into G3P, and disruption of *GLI1* leads to a glycerol-insensitive phenotype [33]. Consistent with previous observations [33], *gli1* plants exhibited no difference in PR length under normal and glycerol treatment conditions (Figure 2B and C). The disruption of *GPDHC1*, which encodes a protein that catalyzes the conversion of dihydroxyl-acetone phosphate (DHAP) into G3P [45], had a relatively weak influence on root growth under normal conditions; however, the effect of glycerol on the root growth of *gpdhc1* plants was stronger compared with its effect on wild-type (WT) plants (Figure 2B and C). FAD-GPDH is a key enzyme involved in the reaction chain that converts glycerol to DHAP and is responsible for catalyzing the conversion of G3P to DHAP [51]. The *fad-gpdh* mutant is unable to survive when seeds are sown directly onto soil; however, it can survive in soil and grow well when it is germinated on half-strength MS medium containing sucrose and later transferred into soil after producing green cotyledons (data not shown). These results agree with the pervious findings [44]. The mutation in *FAD-GPDH* caused severe retardation of root growth, even under normal MS conditions, and the retarded growth was significantly exacerbated by 250 µM glycerol (Figure 2B and C). The *fad-gpdh* plants eventually lost the ability to germinate at 1 mM glycerol (Figure 2B).

The numbers of LRP in WT and mutant plants were further examined. Unlike WT plants, which showed a significant increase in the number of LRP at Stage D in response to an increase in glycerol in the media (Figure 2D), the numbers of LRP at each stage in *gli1* plants were stable under different glycerol concentrations and did not significantly differ from what was observed under normal conditions (Figure 2D). The *gpdhc1* mutant showed a significant decrease in the number of LRP at Stages A and B and an increase in the number of LRP at Stage D with an increase in glycerol concentration (Figure 2D). The LRP development of *fad-gpdh* plants was completely abolished under glycerol treatment due to severely retarded PR growth (data not shown).

Given that all the genes discussed above are involved in G3P homeostasis, the effect of glycerol on root development in the mutants may be due to altered G3P production. Thus, the above data strongly suggest that the effects of glycerol on PR length may have resulted from an imbalance in G3P levels in the plants.

### Impaired Root Growth under Glycerol Treatment is Associated with Increased G3P Accumulation

**Figure 3:**
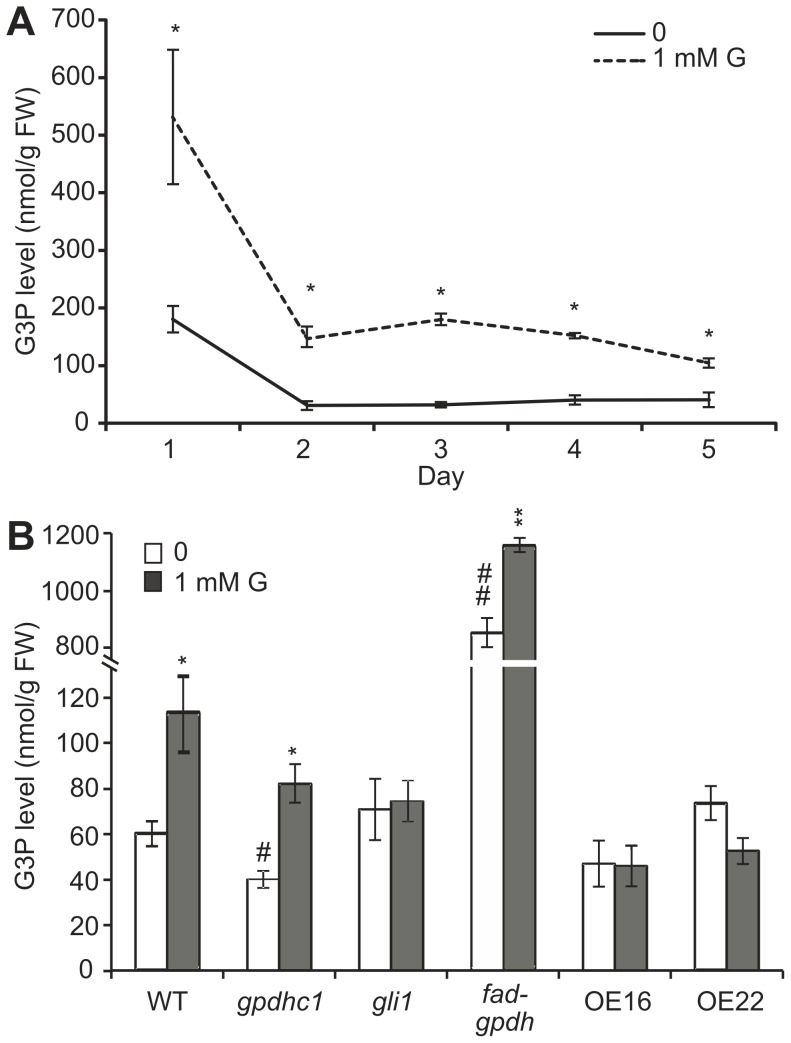
G3P levels in seedlings treated with glycerol (nmol g^−1^ FW). (A) Wild-type seedlings were grown on agar plates containing 0.5×Murashige and Skoog (MS) medium plus 1% (w/v) sucrose in the absence or presence of 1 mM glycerol from 1–5 days post-germination (dpg). The G3P levels of the seedlings were examined. The data are presented as the mean ± SE (n = 3–4). The asterisks indicate significant differences between the means as determined by Student’s t-test (control versus 1 mM glycerol: *, p<0.05; **, p<0.01). (B) Wild-type, *gli1, gpdhc1*, *fad-gpdh*, OE #16 and OE #22 seedlings were grown on agar plates containing 0.5×MS medium plus 1% (w/v) sucrose in the absence or presence of 1 mM glycerol for 4 days. The G3P levels were assayed. The data are presented as the mean ± SE (n = 3–6). The symbols indicate significant differences between the means (*: control versus 1 mM glycerol; #: WT versus mutants and OE lines. *, #: p<0.05; **, ##: p<0.01).

The production of G3P from glycerol is the first step in glycerol catabolism in plants. Because glycerol catabolism-related mutants exhibited different responses to glycerol with respect to root development, the endogenous G3P level may be altered under glycerol treatment. To test this hypothesis, we examined the G3P level in WT plants under glycerol treatment from 1–5 dpg. At 1 dpg, The G3P level of seedlings grown on medium containing 1 mM glycerol was approximately two times higher than that in seedlings grown on control medium (Figure 3A). Although the G3P levels of the plants in both treatment and control groups decreased from 2–5 dpg, the G3P level of plants in the treatment group was significantly higher than that of the control plants at each time point (Figure 3A).

The G3P content in seedlings was further analyzed in *gli1*, *gpdhc1* and *fad-gpdh* mutants as well as in WT in the absence or presence of glycerol. In the absence of glycerol, the G3P content in all plants except the *fad-gpdh* mutant remained at a relatively low level, which is consistent with previous studies [45]. In addition, a high level of G3P in the *fad-gpdh* mutant (Figure 3B) was detected; this is due to a defect in G3P metabolism, as demonstrated previously [44]. Under glycerol treatment, the G3P content of WT seedling was significantly increased by more than 80% (from 60.3±5.5 to 112.6±16.7 nmol/g.FW; Figure 3B), which was consistent with a previous study [42]. The G3P contents remained stable in *gli1* seedlings while increased by 35% and 100% in *fad-gpdh* (from 854.2±51.2 to 1157.6±24.0) and *gpdhc1* (from 40.5±3.8 to 82.3±8.3) mutants seedlings under glycerol treatment, respectively (Figure 3B).

To monitor changes in other related metabolites, we also measured the contents of DHAP and glycerol in WT and *gli1* seedlings after glycerol treatment. From 1 to 5 dpg, the change in the glycerol level in WT seedlings showed a similar pattern to the change in G3P levels (Figure S3A). The glycerol level in *gli1* seedlings was significantly higher than that in WT in normal condition, agreed with previous study [33], and further increased significantly under glycerol treatment (Figure S3B). On the other hand, the DHAP levels in wild-type seedlings did not significantly differ between the treatment and control groups from 1 to 5 dpg (Figure S3C), and the DHAP level in *gli1* seedlings did not significantly differ compared with WT (Figure S3D). These data suggest that exogenously supplied glycerol can enhance the accumulation of endogenous G3P but does not affect DHAP levels in WT plants. Taken together, these results provide evidence that the modulation of root growth observed under glycerol treatment was likely the result of glycerol catabolism in plants.

### Overexpression of the *FAD-GPDH* Gene Ameliorates the Effect of Glycerol on Root Growth

**Figure 4:**
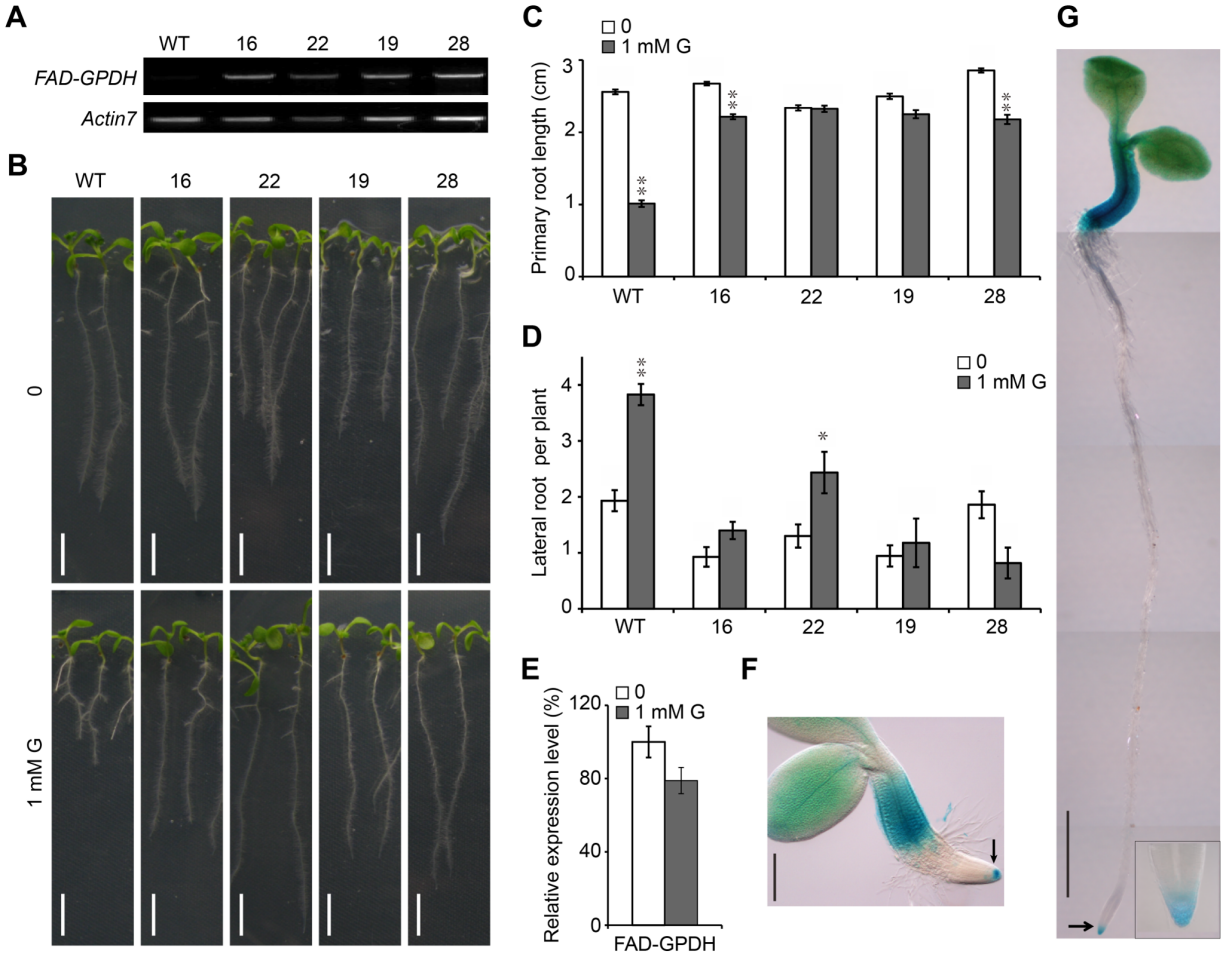
The overexpression of *AtFAD-GPDH* in Arabidopsis ameliorates the effect of glycerol on root development. (A) Transgene expression levels in different transgenic lines. Reverse transcription PCR (RT-PCR) was performed on cDNA made from total RNA extracted from 7-day-old wild-type and *35Spro::AtFAD-GPDH* seedlings. *Actin07* is shown as an internal control. (B) Seven-day-old wild-type seedlings and four *35Spro::AtFAD-GPDH* lines were grown on agar plates containing 0.5×MS medium plus 1% (w/v) sucrose in the presence or absence of 1 mM glycerol. Bar = 0.5 cm. (C and D) Primary root (PR) length (C) and lateral root (LR) number (D) of 7-day-old wild-type and transgenic seedlings described in (B) were recorded. The data are presented as the mean ± SE (n≥20). (E) The relative expression of *FAD-GPDH* in the 8-day-old wild-type seedling roots under 1 mM glycerol treatment compared with untreated control. (F and G) The expression pattern of *FAD-GPDHpro:GUS* in germinating seeds (F); Bar = 100 µm, and 3-day-old seedlings (G) under normal growth conditions; Bar = 500 µm. At least five transgenic plants were observed at each stage, and representative images are presented. The arrows in (F) and (G) show GUS staining in the root cap.

Given that the G3P level in glycerol-treated plants is associated with modifications in root development and both the *gpdhc1* and *fad-gpdh* mutants are more sensitive to glycerol as compared with wild-type plant, we asked whether the overexpression of genes encoding glycerol-3-phosphate dehydrogenase would enhance the tolerance to exogenous glycerol. We produced a large number of transgenic plants expressing a *35Spro::FAD-GPDH* (*FAD-GPDH^OE^*) construct and four other genes (*GPDHp1, GPDHp2/GLY1*, *GPDHc1* and *GPDHc2*) encoding GPDH were used in the experiment for comparison purposes. After a verification of transgenic plants from each construct, at least four transgenic lines with obviously increased transgenic expression were assayed for their primary root growth performance under glycerol treatment. Except the transgenic plants expressing *35Spro::FAD-GPDH* (Figure 4), none of other transgenic lines showed a significant difference in their glycerol tolerance compared with WT (Figure S4). Therefore, in the subsequent analysis, four stable *FAD-GPDH^OE^* T_4_ lines (#16, 22, 19 and 28) that exhibited an obvious increase in *FAD-GPDH* expression were selected for further analysis (Figure 4A).

The inhibitory effects of a 7-day glycerol treatment on PR length in the *FAD-GPDH^OE^* lines were completely reversed (OE #19 and OE #22) or significantly weakened (OE #16 and OE #28) when compared with WT (Figure 4B and C), suggesting that much elevated *FAD-GPDH* expression in the transgenic plants (Figure 4A) can significantly increase the tolerance to glycerol. We noticed that there were some variations in the PR length among the transgenic lines, which is understandable as the transgenic expression levels may not be exactly same among the different transgenic lines (Figure 4A). After glycerol treatment, smaller increases in the numbers of LRs in the transgenic lines were consistently observed (0.25- to 0.8-fold) compared with WT, in which the number of LRs nearly doubled (Figure 4D). This finding clearly demonstrated that the overexpression of *FAD-GPDH* can minimize the effect of glycerol on root growth and development.

We also measured the G3P contents of two *FAD-GPDH^OE^* lines (OE#16 and OE#22). Coinciding with the increased glycerol tolerance, the level of G3P in two *FAD-GPDH^OE^* lines was not significantly different from the untreated plants (Figure 3B), suggesting that the overexpression of *FAD-GPDH* have reduced the accumulation of endogenous G3P and ameliorated the effect of glycerol on root growth. On the other hand, the levels of DHAP and glycerol in the two *FAD-GPDH^OE^* lines were significantly increased compared with WT (Figure S3C–D). We also tested the effect of exogenous application of 1 mM G3P on WT, the mutants and the *FAD-GPDH^OE^* lines. The PR length of WT was reduced under exogenous G3P treatment; however, the PR lengths of the *gli1* mutant and the *FAD-GPDH^OE^* lines did not change or decreased slightly (Figure S5). Taken together, these data suggest that increased tolerance to glycerol and G3P in the *FAD-GPDH^OE^* lines may be due to the consumption of G3P as a result of the elevated FAD-GPDH level.

Quantitative RT-PCR (qRT-PCR) analysis of *FAD-GPDH* expression in WT plants exposed to 1 mM glycerol revealed a 22% decrease, which did not reach statistical significance (Figure 4E), suggesting that the expression level of *FAD-GPDH* could be important for root development under glycerol treatment. To investigate the expression pattern of the *FAD-GPDH* gene, a 1350-bp promoter region upstream of the start codon of *FAD-GPDH* was fused with the *β*-glucuronidase (GUS) reporter gene. Germinating seeds from independent transgenic Arabidopsis lines were analyzed. Strong GUS staining was observed in the root cap as well as the hypocotyl and the cotyledon (Figure 4F), which was consistent with previous observations [51]. Interestingly, the only predominant staining observed in the root occurred at the root tip (marked with arrows and a black box in Figure 4G), which coincided with *FAD-GPDH* abundance at root tip (based on data from the eFP browser database; Figure S6).

### Overexpression of *FAD-GPDH* Prevented the Reduction of Pi Levels in Roots under Glycerol Treatment

**Figure 5:**
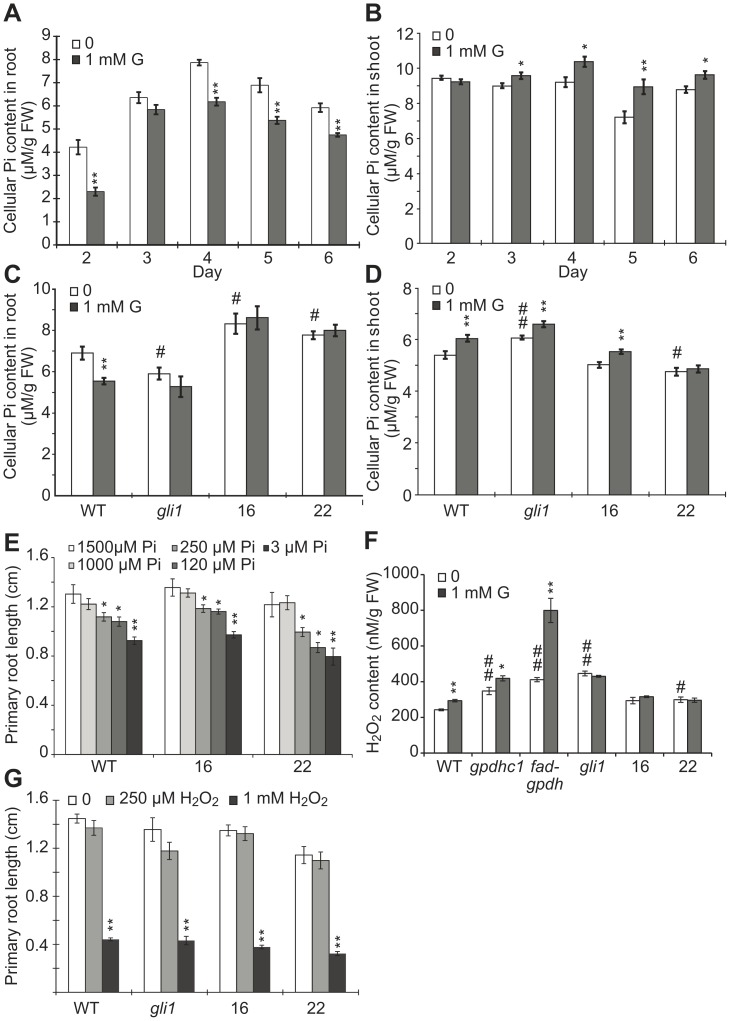
Pi and H_2_O_2_ levels in seedlings treated with glycerol. Wild-type seedlings were grown on agar plates containing 0.5×MS medium plus 1% (w/v) sucrose in the absence or presence of 1 mM glycerol from 2–6 days post-germination (dpg). (A–B) The cellular Pi level (µmol g^−1^ FW) was analyzed in wild-type roots (A) and shoots (B) for the indicated number of days. The data are presented as the mean ± SE (n = 4–6). (C–D) The cellular Pi level in the roots (C) and shoots (D) of the *gli1* mutant, OE #16, OE #22 and wild-type seedlings grown on 0 or 1 mM glycerol medium was assayed at 4 dpg. Values are expressed as the mean ± SE (n = 4). (E) The effect of phosphate availability on the primary root (PR) length in wild-type and *FAD-GPDH^OE^* lines. Arabidopsis wild-type (Col-0), OE #16 and OE #22 seedlings were grown on the surface of agar plates containing various concentrations of phosphate for 6 days, and the PR length was measured. (F) Quantification of H_2_O_2_ in *gli1, gpdhc1* and *fad-gpdh* mutants, OE #16, OE #22 and wild-type seedlings grown on 0 or 1 mM glycerol medium was performed at 5 dpg. The values are expressed as the mean ± SE (n = 4). (G) The effect of exogenous H_2_O_2_ on PR length. PR lengths of 5-day-old *gli1* mutants, OE #16, OE #22 and wild-type seedlings in the presence of 0, 250 µM and 1 mM H_2_O_2_ were recorded. Values are expressed as the means ± SE (n = 18–20). Different symbols indicate that the means differ significantly by Student’s t-test (*: control versus 1 mM glycerol or other treatment; #: WT versus mutants or OE lines. *, #: p<0.05; **, ##: p<0.01).

Previous studies have shown that the application of glycerol can reduce the phosphate pool [35] and that a low level of available phosphate inhibits PR length and promotes LR development [22], [52]. To test the effect of glycerol on phosphate availability, we examined the cellular Pi content in plants treated with 1 mM glycerol. The Pi content in the roots of glycerol-treated WT plants was significantly lower than that in untreated controls at most time points examined (Figure 5A), while the Pi level in the shoots of glycerol-treated WT plants was significantly higher than the control from 3 to 6 dpg (Figure 5B). On the other hand, the Pi content in the roots of the glycerol-treated *gli1* mutant and the *FAD-GPDH^OE^* lines (OE #16 and OE #22) appeared similar to that of the untreated control (Figure 5C). Furthermore, the Pi levels in the glycerol-treated shoots of *gli1* and the two transgenic lines were increased with more significant changes in OE #16 than in OE #22 (Figure 5D). The difference between the two transgenic lines may be due to different *FAD-GPDH* transgenic expression levels or some possible physiological variations. Interestingly, the root Pi level in *gli1* was lower compared with wild-type plants in normal conditions (Figure 5C), while the shoot Pi level in *gli1* was higher as compared with wild-type plant (Figure 5D). On the other hand, the root Pi levels of WT and *gli1* plants after glycerol treatment were similar, but the shoot Pi level in *gli1* plants was higher than in WT plants, indicating that the root phenotype might be not similar to the shoot under glycerol treatment.

We next tested the effect of exogenous low phosphate availability on PR length and found that the PR length of the seedlings did not significantly differ when the Pi level was reduced from 1.5 mM to 1 mM; however the PR lengths of OE #16, OE #22 and WT were significantly reduced when the Pi availability was decreased to 250 µM or below (Figure 5E). The above data suggested that glycerol treatment affected the Pi availability in WT plants and resulted in a reduction of cellular Pi in roots, which may contribute to the altered development of roots under the treatment together with other factors. Taken together, the data from above experiments suggested that there may have a relationship between the endogenous G3P metabolism and phosphorous availability.

### The Glycerol-induced Increase in H_2_O_2_ Content was Abolished in *FAD-GPDH^OE^* Seedlings

Because impaired glycerol metabolism results in elevated ROS levels in plant cells [45], it is possible that ROS homeostasis regulated by the G3P shuttle might contribute to root development. To understand the relationship between root development and the altered ROS levels induced by exogenous glycerol, we investigated the potential role of H_2_O_2_ in glycerol-dependent inhibition of root growth because H_2_O_2_, as a ROS, has been found to be associated with the alteration of plant growth under various stresses [17]–[19]. Compared with WT, the basal levels of H_2_O_2_ in the *gpdhc1, fad-gpdh* and *gli1* mutant were increased significantly by 43%, 69% and 84%, respectively (Figure 5F), which is consistent with findings from previous studies [45]. The basal H_2_O_2_ levels in both *FAD-GPDH^OE^* lines were increased with a higher level in OE#22 line (Figure 5F). After glycerol treatment, the H_2_O_2_ level in WT, *gpdhc1* and *fad-gpdh* plants was elevated significantly (by 21%, 20% and 94%, respectively) compared with that in untreated plants (Figure 5F). The H_2_O_2_ levels in the *gli1* mutant, OE#16 and OE #22 remained unchanged significantly as compared with the untreated plants (Figure 5F). The above data thus suggest that the inhibition of PR length under glycerol treatment may be partially related to the increased H_2_O_2_ level. However, treatment of WT, *gli1* and OE lines #16 and #22 with exogenous H_2_O_2_ had similar inhibitory effects on PR length (Figure 5G); this result implies that the glycerol-induced increase in endogenous H_2_O_2_ might elicit a different response compared with that induced by exogenous H_2_O_2_ stress.

### Glycerol Exerted Similar Inhibitory Effects on the PR Growth of WT and *act1* Mutant Plants

Previous studies have shown that exogenous application of 50 mM glycerol lowers oleic acid levels in a G3P acyltransferase (ACT1)-dependent manner [37], [38] and induces nitric oxide (NO) accumulation in Arabidopsis plants [53]. We therefore tested the possibility that glycerol application induces defense responses by lowering oleic acid levels, which could be responsible for the altered root phenotype in glycerol-treated plants [37], [38]. To accomplish this, we compared the responses of WT and *act1* mutant (Salk_069657 and CS200) plants to exogenous glycerol by examining their fatty acid levels and PR lengths. As in previous reports [54], [55], both Salk_069657 and CS200 mutants showed obviously reduced hexadecatrienoic acid levels and increased oleic acid levels (Figure S7A). Interestingly, the fatty acid levels of both WT and the mutants did not change significantly between the control and the 1 mM glycerol treatment (Figure S7A). Both *act1* and WT plants showed an obvious reduction in PR length after the application of 1 mM glycerol (Figure S7B). The above results suggest that the effects of glycerol on root growth may not be related to the oleic acid-mediated signaling pathway and perhaps the effect of glycerol on fatty acid level could be concentration dependent.

### Glycerol Affects Endogenous IAA Content and Auxin Distribution at the Root Tip

**Figure 6:**
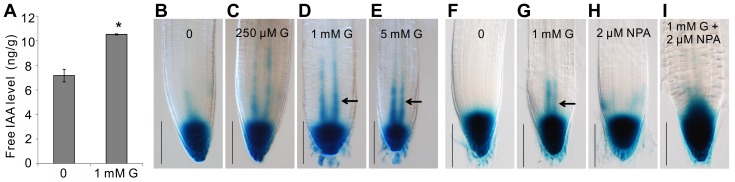
Endogenous IAA content and auxin distribution in the root tip were altered under glycerol treatment. (A) Free IAA content in 6-day-old wild-type seedlings grown on 0.5×MS media plus 1% sucrose with or without 1 mM glycerol. The data are presented as the mean ± SE (n = 4). Asterisks (*) represent significant differences (p<0.05) by Student’s t-test. (B–E) *DR5pro:GUS* staining in 6-day-old seedlings grown on 0.5×MS medium containing various concentrations of glycerol (0 [B], 250 µM G [C], 1 mM G [D] and 5 mM G [E]). Photographs are representative of at least five stained plants. Bar = 100 µm. (F–I) *DR5pro:GUS* staining in seedlings grown on 0.5×MS medium containing various concentrations of glycerol and/or NPA (untreated control [F], 1 mM glycerol [G], 2 µM NPA [H] or 1 mM glycerol and 2 µM NPA [I]). *DR5pro:GUS* plants were grown on 0.5×MS medium for 4 days and subsequently transferred to medium with various additives for another 2 days of growth. The seedlings were then sampled for β-glucuronidase (GUS) staining. Bar = 100 µm.

Because we observed that exogenous glycerol triggered root architecture remodeling, we questioned how this effect might be perceived during root development. Auxin has been shown to exert major effects on root development; therefore, we measured the free IAA content in WT roots treated with 1 mM glycerol to investigate whether root growth is mediated by an auxin-related pathway under this treatment. We found that the free IAA level in roots under glycerol treatment was significantly increased by 46% (from 7.2 to 10.5 ng/g fresh weight [FW]; Figure 6A).

Given that the accumulation of IAA under glycerol treatment may be caused by alterations in auxin biosynthesis or auxin transport, we monitored the expression of the synthetic auxin-responsive element *DR5* [56]. Normally, the expression of *DR5-GUS* can be observed in the quiescent center (QC) and the root cap (Figure 6B and F) [56]. An increased level of GUS staining was observed in the stele cells of the root meristem under glycerol treatment (Figure 6C-E and G), indicating that normal auxin distribution was altered.

**Figure 7:**
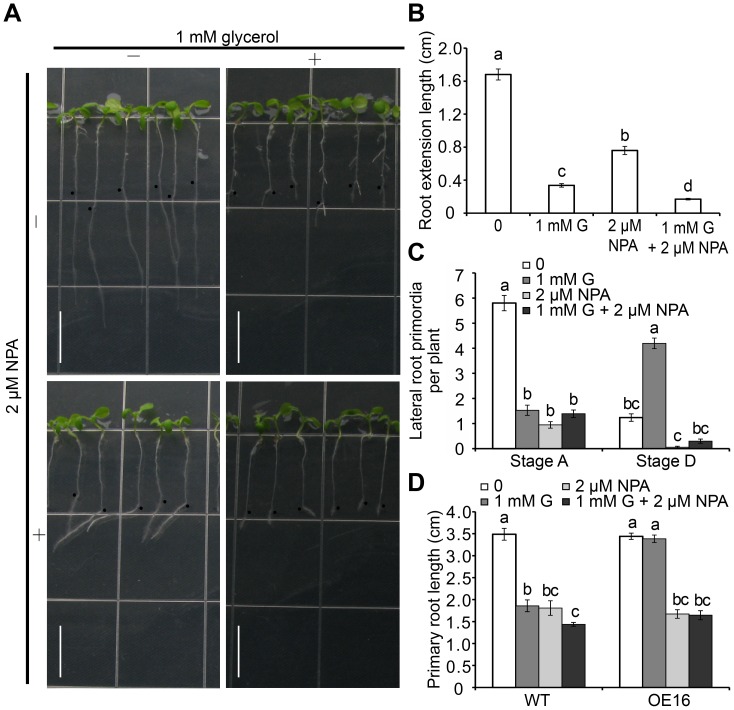
The effect of auxin transport inhibitor NPA on root development in Arabidopsis seedlings. (A) Growth performance of wild-type seedlings under glycerol and NPA treatments. Wild-type seedlings were grown on 0.5×MS medium for 5 days and subsequently transferred to media containing 1 mM glycerol with or without 2 µM NPA for 3 days. Black dots indicated the starting growth positions of the PR tip after shift. Bar = 1 cm. (B) Primary root (PR) extension lengths and (C) Lateral root primordia (LRP) at Stages A and D in wild-type plants grown under the conditions as described in (A) were recorded. The data are presented as the mean ± SE (n = 36). (D) Comparison of wild-type and OE #16 plants under glycerol and NPA treatments. Wild-type and OE #16 seeds were sown directly onto media containing 0 mM glycerol, 1 mM glycerol and 2 µM NPA or 1 mM glycerol and 2 µM NPA for 7 days; the root lengths of the plants exposed to each treatment were then measured (n≥27). Different letters indicate significant differences (p<0.05).

*N*-naphthylphthalamic acid (NPA), an auxin efflux inhibitor, inhibits polar auxin transport from shoots to roots and impairs root growth [57]. The increased expression of *DR5-GUS* in stele cells under glycerol treatment was inhibited by 2 µM NPA (Figure 6H–I). NPA alone inhibited root growth; however, this inhibition was less dramatic compared with that observed when glycerol was applied alone (Figure 7A and B). When both NPA and glycerol were included in the medium, the inhibition of root growth was more severe than that observed with NPA or glycerol alone (Figure 7A and B), suggesting that glycerol and auxin might exert overlapping and/or different effects on PR length.

Furthermore, the effect of NPA on LRP was investigated at two stages (A and D). Compared with the untreated control, LRP formation was severely inhibited in the presence of glycerol or NPA alone (Figure 7C), which was consistent with previous reports [22]. No significant difference was observed between the single and combined treatments (Figure 7C). Stage D LRs were reduced by 95% in the presence of 2 µM NPA. Additionally, the glycerol-induced increase in the number of LRs was reversed by the addition of NPA (from 4.2 to 0.3 LRs per plant). Taken together, these data indicate that glycerol likely induces LR formation through the regulation of polar auxin transport.

The *FAD-GPDH* ^OE^ lines were found to tolerate glycerol (Figure 4). This characteristic was further evaluated under NPA treatment with one representative transgenic line (OE #16). Exposure to NPA alone caused a reduction in root length that was similar between OE #16 and WT seedlings, although a further decrease was not observed with a combination of glycerol and NPA (Figure 7D), indicating that the *FAD-GPDH* ^OE^ plant is tolerant to glycerol, but not NPA.

### Glycerol Treatment Alters the Expression of *PIN1* and *PIN7*

**Figure 8:**
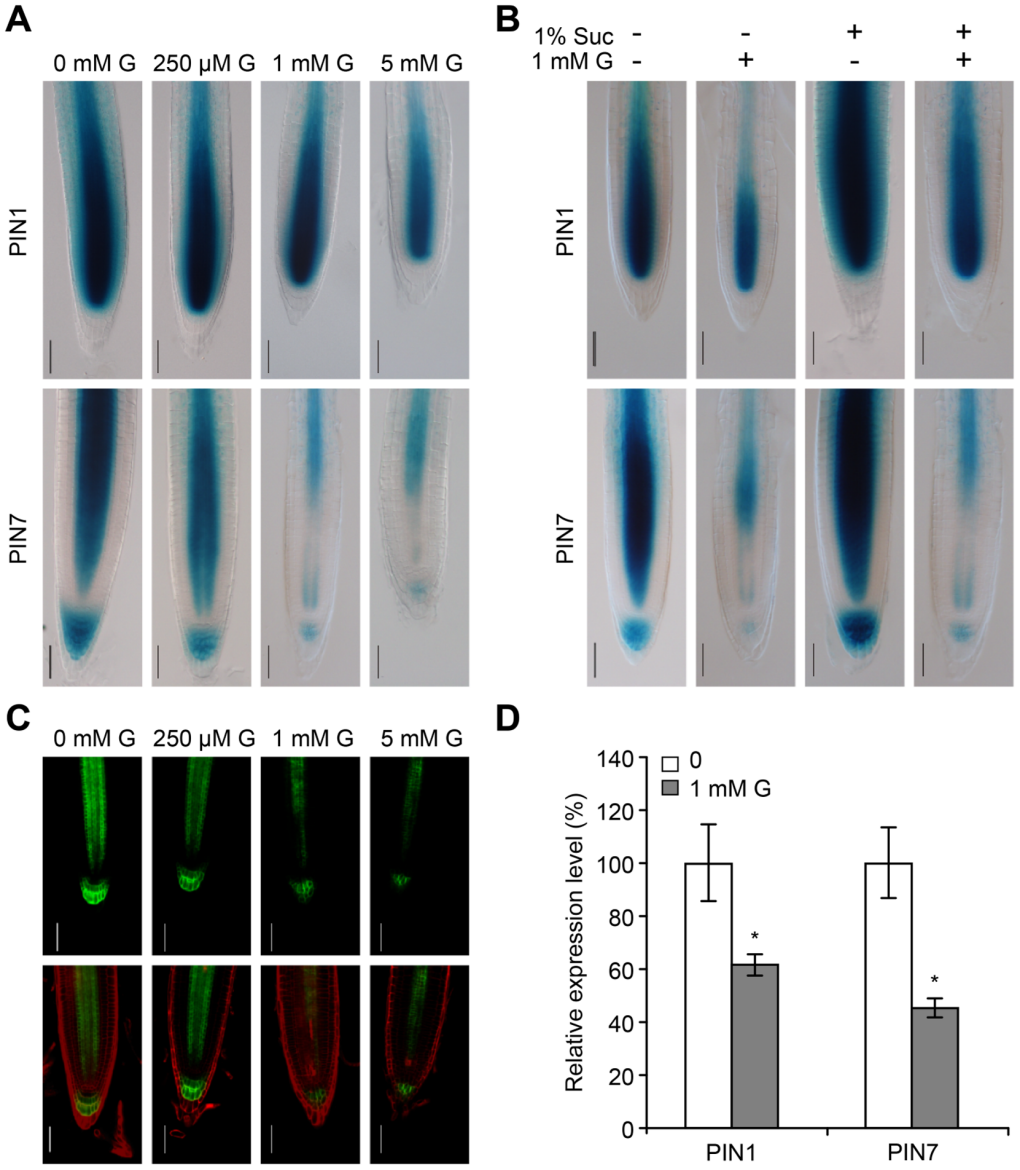
Auxin transport-related genes were analyzed by GUS staining, confocal microscopy and qRT-PCR. (A) Staining patterns of *PIN1pro::GUS* (upper panels) and *PIN7pro::GUS* (lower panels) under glycerol treatment. Seeds were germinated and grown on 0.5×MS medium with various concentrations of glycerol (0, 250 µM, 1 mM and 5 mM) for 6 days and subjected to GUS staining. The micrographs are representative of at least five stained plants from each treatment. Bar = 50 µm. (B) *PIN1pro::GUS* and *PIN7pro::GUS* seedlings were grown on media with or without 1% sucrose in absence or presence of 1 mM glycerol for 6 days and subjected to GUS staining. Bar = 50 µm. (C) *PIN7pro::PIN7-GFP* expression in the roots of 5-day-old seedlings exposed to various concentrations of glycerol (0, 250 µM, 1 mM and 5 mM). GFP images were recorded by confocal microscope. Bar = 50 µm. (D) *PIN1* and *PIN7* expression in 6-day-old wild-type roots. The data are presented as the relative expression under 1 mM glycerol treatment compared to the untreated control. Asterisks (*) indicate significant differences at P<0.05 by Student’s t-test.

Polar auxin flow is extremely important for the establishment and maintenance of auxin gradients, and this process requires transport facilitators of the PIN family that exhibit polar localization [6]. To understand whether the changes in auxin flow under glycerol treatment were mediated by PIN proteins, we observed the expression patterns of *PIN1pro::GUS* and *PIN7pro::GUS*, which are two marker genes for auxin gradient patterning [58]. The intensity of *PIN1pro::GUS* staining was reduced at the root apical meristem and the root cap upon exposure to high concentrations of glycerol, and the reduction in *PIN7pro::GUS* staining upon glycerol treatment was even more obvious (Figure 8A). The addition of 1 mM glycerol appeared to reduce the intensities of *PIN1pro::GUS* and *PIN7pro::GUS* staining in the presence or absence of sucrose, and the addition of 1% sucrose appeared to increase expression levels of *PINs* to various extents in the presence or absence of glycerol (Figure 8B). Consistent with the *PIN7pro::GUS* staining, glycerol treatment caused a significant reduction in *PIN7pro::PIN7-GFP* expression (Figure 8C), suggesting that PIN7 distribution was likely modified under glycerol treatment. The expression levels of *PIN1* and *PIN7* under glycerol treatment were significantly decreased to 61% and 45% of the untreated control, respectively (Figure 8D). Thus, our data suggest that glycerol affects polar auxin flow in roots.

### *TIR1* and *ARF7* are Important for Controlling LR Development in Response to Glycerol

**Figure 9:**
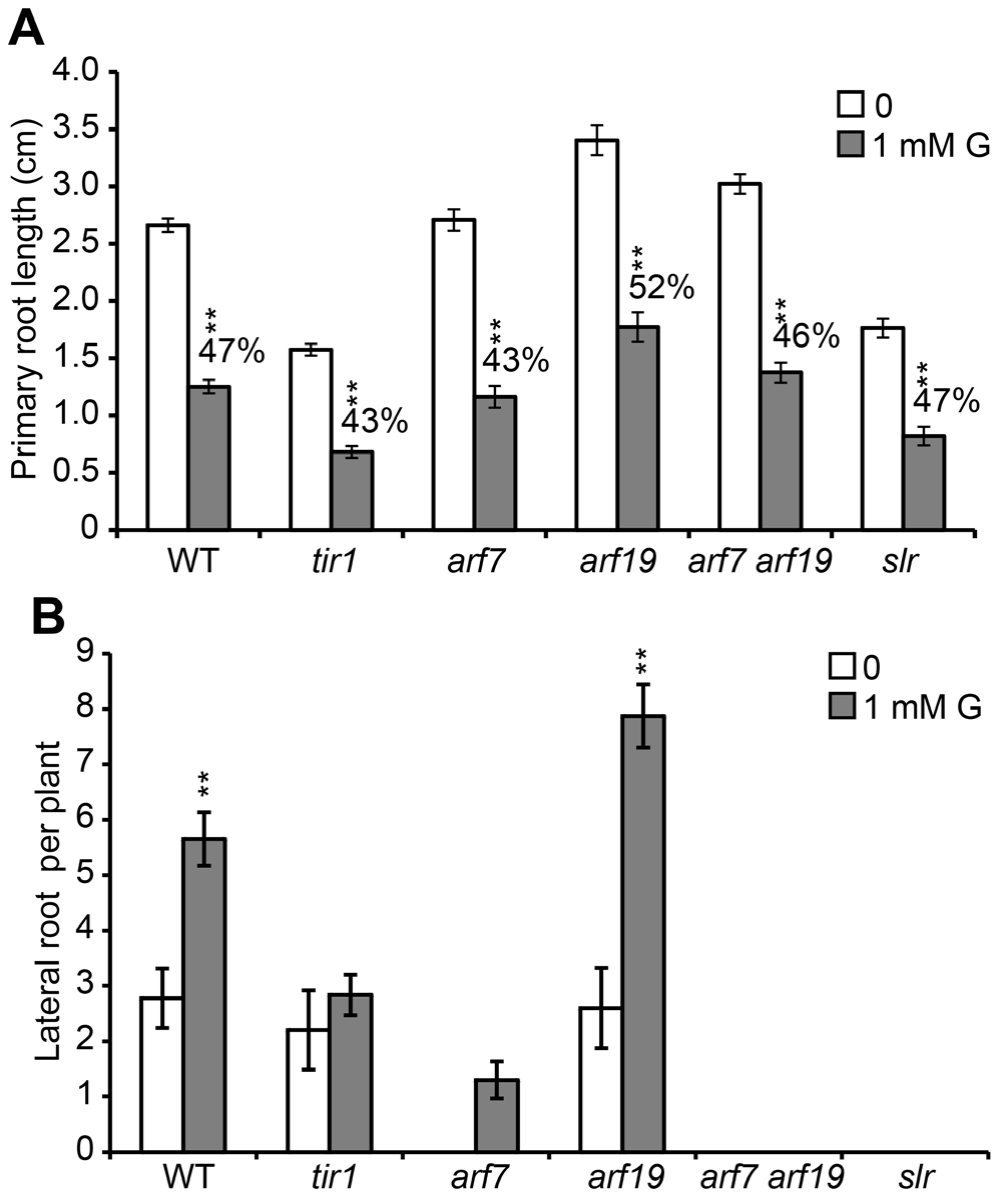
Comparison of the root growth of auxin-related mutants grown in the presence of glycerol. Primary root (PR) length (A) and lateral root (LR) number per plant (B) are shown for WT, *tir1*, *arf7*, *arf19*, *arf7 arf19* and *slr* plants. All the plants were grown on 0.5× Murashige and Skoog (MS) medium containing 1% sucrose for 4 days and subsequently transferred to media with or without 1 mM glycerol for 4 days. The values shown are the mean of 9 seedlings. Asterisks indicate significant differences from the control based on Student’s t-test (*, P<0.05; **, p<0.01).

To understand whether the promotion of LRs is dependent on PR inhibition under glycerol treatment, we next analyzed several mutants with disruptions in genes involved in auxin signaling and LR development. Under treatment with 1 mM glycerol, *tir1*, *arf7, arf19* and *slr* plants showed similar reductions in PR length (43%–52%) compared with WT (47%) (Figure 9A). However, the LR development of the mutants varied greatly. The number of LRs in *tir1* did not change significantly (Figure 9B), suggesting that TIR1 could play a role in modulating root architecture in response to glycerol. The number of LRs in *arf19* increased significantly under glycerol treatment, as it did in WT plants; however, the LR number in *arf7* only marginally increased (Figure 9B), and the PR length of *arf7* was reduced dramatically under glycerol treatment (Figure 9A). Moreover, no LR formation was observed in the *arf7arf19* double mutant or the *slr* mutant (Figure 9B). Taken together, these data suggest that *TIR1* and *ARF7* are involved in the establishment of root architecture, including increased LR formation, in response to glycerol.

### Exogenous Glycerol Decreases the Meristem Cell Number and Downregulates Cell Cycle Gene Expression

**Figure 10:**
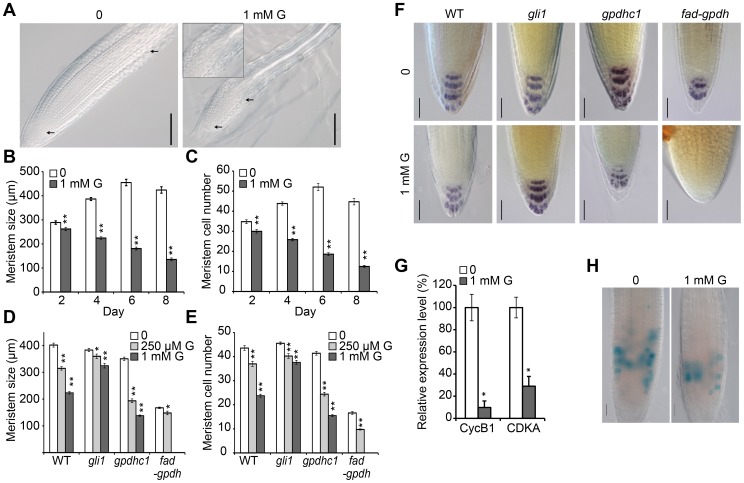
Root meristem cell and cell cycle gene expression in glycerol-treated seedlings of wild-type and mutants. The seedlings were grown on 0.5×MS medium plus 1% sucrose with or without 1 mM glycerol. (A) Nomarski images showed the meristems of wild-type seedlings gown on 0.5×MS medium in the absence (left) or presence (right) of 1 mM glycerol at 8 dpg. Arrows mark the boundaries of the meristem region. Bars = 100 µm. The meristem size (B) and meristem cell number (C) of wild-type plants grown on media with or without glycerol at different developmental stages were investigated. Meristem size (D) and meristem cell number (E) of wild-type, *gpdhc1*, *gli1* and *fad-gpdh* seedlings grown for 7 days on 0, 250 µM and 1 mM glycerol media were recorded. The data are presented as the mean of 30–40 seedlings ± SE. (F) Starch granules in wild-type, *gli1*, *gpdhc1* and *fad-gpdh* seedlings were visualized by Lugol staining in the presence or absence of 1 mM glycerol. Four-day-old seedlings were first fixed in FAA at 4°C overnight and subsequently washed once in 50% ethanol. The samples were then placed in Lugol solution (0.37% iodine and 0.71% potassium iodide) for 1 min and transferred to a chloral hydrate solution for 2 min. The micrographs are representative of at least 10 seedlings for each genotype. Bars = 20 µm. (G) The expression levels of *CycB1* and *CDKA* in the root tip at 8 dpg were analyzed under glycerol treatment. (H) Seedlings expressing *CycB1;1-GUS* were grown on media in the presence or absence of 1 mM glycerol for 6 days and subjected to GUS staining. Bar = 10 µm. Asterisks indicate that the means differ significantly from the control by Student’s t-test (*, p<0.05; **, p<0.01).

To understand the cytological basis of altered root development under glycerol treatment, we examined the size and cell number of the root meristem by surveying the cells in the cortex layer from the QC to the start of the elongation zone (Figure 10A). The root meristem size of glycerol-treated seedlings was significantly smaller compared with that of the seedlings grown under control conditions at 2 dpg (Figure 10B). This difference became more significant with extended treatment, and a 1∶3 ratio for meristem size between treated and untreated plants was reached at 8 dpg (Figure 10A and B). Similarly, the number of meristem cells also decreased significantly under glycerol treatment at all the time points examined (Figure 10C).

The root meristem sizes and meristem cell numbers of *gli1*, *gpdhc1* and *fad-gpdh* roots in media containing different concentrations of glycerol were quantified. In general, both the meristem size and meristem cell number decreased gradually with increasing concentrations of glycerol; however, the extent of the reduction varied depending on the genotype of the plant (Figure 10D and E). For example, the changes in meristem size and cell number in *gli1* mutants were less dramatic than those observed in WT and *gpdhc1* plants. *fad-gpdh* exhibited abnormal growth even under control conditions; however, the changes in root meristem size and cell numbers in this mutant under 250 µM glycerol treatment were relatively small compared with the changes observed in plants of the other genotypes (Figure 10D). Furthermore, Lugol staining analysis revealed that the number of columella cell layers was reduced under glycerol treatment in WT, *gpdhc1* and *fad-gpdh*, as indicated by the starch granule accumulation pattern (Figure 10F). Taken together, these data suggest that the glycerol-dependent inhibition of root length may be attributed to a decrease in meristem size and cell number.

Changes in meristem cell number under glycerol treatment (Figure 10B and C) indicate that exogenous glycerol stress might influence cell cycle progression. To test this hypothesis, we analyzed the expression of *CYCB1;1* and *CDKA*, two important marker genes involved in cell cycle control. qRT-PCR analysis revealed that both genes were significantly downregulated under glycerol treatment (p<0.05) (Figure 10G), suggesting that the cell cycle may be inhibited under this condition. This finding was further supported by *CYCB1;1pro::GUS* staining, which revealed a decrease in the number of root meristem cells under glycerol treatment (Figure 10H).

## Discussion

The exogenous application of glycerol to plants has multiple effects on several important cellular processes. The biochemical reactions that occur during glycerol metabolism are well known; however, the details regarding how glycerol affects plant growth from a developmental point of view are poorly understood. The root is a critical organ of higher plants and is also useful as a model system for developmental biology studies. The current study showed that root system architecture was modified under exogenous glycerol treatment in Arabidopsis. Overexpression of the *FAD-GPDH* gene increased the ability of transgenic plants to tolerate exogenous glycerol stress. We showed that several factors, such G3P, phosphate, ROS and auxin, may contribute to the effects of glycerol on root growth.

**Figure 11:**
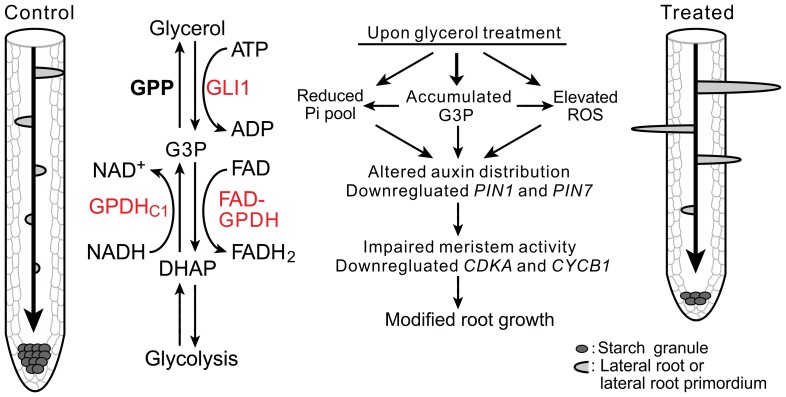
A model illustrating glycerol-triggered modulation of root development. The diagram shows the different root patterns in the absence (left) or the presence (right) of glycerol. The middle section shows a condensed schematic of plant glycerol metabolism and three important genes in this study (red). Glycerol is phosphorylated to G3P by GLI1 and can also be generated by GPDHc1 via the reduction of DHAP. G3P is oxidized to DHAP by FAD-GPDH or dephosphorylate to glycerol by GPP. Exogenous glycerol treatment can cause modifications of multiple pathways, including increased G3P and reactive oxygen species (ROS) levels, reduced the phosphate level and expression of *PIN1* and *PIN7*. It also affected polar auxin transport and the root meristem activity, thus resulting in modified root growth and development. Abbreviations: GLI1, glycerol kinase; GPDHc1, cytosolic glycerol-3-phosphate dehydrogenase; FAD-GPDH, flavin adenine dinucleotide-dependent glycerol-3-phosphate dehydrogenase; GPP, glycerol-3-phosphatase; G3P, glycerol-3-phosphate; DHAP, dihydroxyacetone phosphate; ATP, adenosine triphosphate; FAD, flavin adenine dinucleotide; NADH, the reduced form of nicotinamide adenine dinucleotide. For the sake of clarity, some co-substrates and/or co-products have been omitted in some reactions.

G3P levels are maintained by glycerol kinase and FAD-GPDH. Glycerol kinase, which phosphorylates glycerol to generate G3P and consumes ATP simultaneously (Figure 11), plays an essential role in the utilization of glycerol in plant cells. A mutant with a disruption in the *GLI1* gene (*gli1*) is unable to catalyze the conversion of glycerol to G3P [33], [59]. We consistently failed to observe a glycerol-induced increase in the G3P level in the *gli1* mutant in this study (Figure 3B). As a result, there was no difference in the PR length or the LR number in *gli1* mutant plants under glycerol treatment (Figure 2). FAD-GPDH oxidizes G3P to generate DHAP (Figure 11) [51]. The G3P level in *fad-gpdh* plants was increased significantly under glycerol treatment, which in turn resulted in a more dramatic reduction of the PR length in the *fad-gpdh* mutant compared with wild-type and other glycerol metabolism-related mutants (Figure 2). In contrast, there was no significant increase in G3P in *FAD-GPDH* ^OE^ plants grown on medium containing glycerol versus control medium, suggesting that the increased FAD-GPDH in cells may convert glycerol to DHAP more rapidly (Figure 3B, Figure S3C). Through this conversion, the effect of glycerol on root growth and development was largely alleviated or reversed (Figure 4C and D). Furthermore, exogenous G3P or glycerol treatment affected PR length similarly in WT, *gli1* and *FAD-GPDH* ^OE^ lines (Figure S5). These results thus illustrate that the G3P level in plant cells has the potential to influence the development of Arabidopsis roots, and increased tolerance to exogenous glycerol can be achieved through the overexpression of *FAD-GPDH*.

A study on sycamore cells revealed that the application of 50 mM glycerol results in a decrease in the orthophosphate level [35]. The phosphate factor can significantly alter root architecture in Arabidopsis [26], [52]. In our study, the Pi level in glycerol-treated root of WT plants from 2 to 6 dpg was significantly reduced compared with the untreated control (Figure 5A), while the Pi levels in the glycerol-treated shoots of WT plants were increased from 3 to 6 dpg compared with the control (Figure 5B), suggesting that the root and shoot phenotypes of glycerol might not be similar. A previous study suggested that the alterations in the pattern of LR emergence and formation in response to Pi availability are mediated by changes in auxin sensitivity and the modulation of auxin sensitivity by Pi was found to be dependent on TIR1 and ARF19 [22]. In the current study, the root IAA content increased under glycerol treatment (Figure 6A), and LR formation was mainly affected by ARF7 rather than ARF19 (Figure 9B). Additionally, the PR length reduction was similar between the *FAD-GPDH^OE^* lines and WT in the presence of 3 µM to 1.5 mM phosphate (Figure 5E). It is possible that the overexpression of FAD-GPDH restores the reduced phosphate pool under glycerol treatment through effects on the G3P shuttle, which may regulate oxidative phosphorylation and ATP production. However, this metabolic signaling pathway may be different from the one that is triggered in response to low exogenous phosphate.

Glycerol-3-phosphate (G3P) transporter is a member of organic phosphate/inorganic phosphate (Pi) anti-porters, which has a higher affinity with G3P than Pi [60], [61]. Differential expression of glycerol-3-phosphate permease (G3Pps) between roots and leaves is observed, implying these transports may involve Pi mobilization from root to leaves [62]. It is possible that Pi distribution works differently in *gli1* mutant and OE lines between shoot versus root. First, root Pi content was low in *gli1* mutants and high in *FAD-GPDH* ^OE^ lines compared with WT (Figure 5C). Second, shoot Pi content was high in *gli1* mutants and low in two OE lines compared with WT (Figure 5D). Third, the effect of glycerol on root Pi content was abolished in *gli1* mutants and both OE lines (Figure 5C). However, the effect of glycerol on shoot Pi content in the *gli1* mutant and the two transgenic lines exhibited a similar trend to WT with more significant changes in OE #16 than in OE #22 (Figure 5D). Overall, the main finding here is that the effect of glycerol on root Pi content in WT is obviously affected by the alteration of *GLI1* and *FAD-GPDH.* The expression level of *GLI1* increases significantly during seed germination and leaf senescence [33]. Thus, phosphate could potentially be recycled from older leaves to new ones via the sequential action of GLI1 and FAD-GPDH. Furthermore, the expression of *FAD-GPDH* in cotyledons appeared more abundant than that in the root base on the GUS staining (Figure 4F–G). It is possible that glycerol metabolism functions differently in the shoot versus the root. Further studies are needed to have a better understanding of such possible difference.

Glucose affects nearly all aspects of root development such as primary root growth, lateral root development and root hair formation [23]. It is well known that both DHAP and glucose-6-phosphate (G6P) are important intermediates in glucose metabolism. In this work, accumulated G3P could not be converted to more DHAP in WT seedlings (Figure S3A). The gene expression of *FAD-GPDH* was not increased under glycerol treatment (Figure 3E). Other studies have reported that G3P dehydrogenase activity in chicory leaf tissue was not significantly different in the presence or absence of glycerol [39]. It is known that G3P converted by GLI1 from glycerol is widely utilized in plant cell metabolism. On the other hand, accumulated G3P would impair several pathways. It is possible that the generation of DHAP via FAD-GPDH is converted to G3P via a glycerol intermediate. In the G3P shuttle, GPDHc1 consumes DHAP to generate G3P, but it is still unclear how G3P is converted to glycerol. Recently, *GPP1* and *GPP2* were found to encode glycerol-3-phosphatases, which were thought to dephosphorylate G3P to glycerol in *Arabidopsis* and yeast [63], [64]. A high level of G3P competitively inhibits the activity of the glycolytic enzyme phosphoglucose isomerase, preventing gluconeogenic flux to sugars by blocking fructose-6-phosphate conversion to glucose-6-phosphate [44]. However, the addition of glycerol to low-sucrose medium also inhibited PR growth and promoted LR development (Table S2). Furthermore, the GUS staining of plants expressing *PIN1pro::GUS* and *PIN7pro::GUS* grown in media without sucrose was also reduced under glycerol treatment compared with the untreated control (Figure 8B). These results suggest that the mechanism by which glycerol alters root architecture could be partially independent of sucrose.

Cellular redox homeostasis has been shown to affect root growth [20], [21]. Shen *et al.* reported that the G3P shuttle adjusts the intracellular redox state and the NADH/NAD^+^ ratio, which involves the combined actions of cytosolic NAD^+^-dependent GPDH and FAD-GPDH [45]. In this study, we showed that accumulated G3P may impair redox exchange and increase H_2_O_2_ production in WT plants under glycerol treatment. The basal H_2_O_2_ levels in the *gpdhc1, fad-gpdh* and *gli1* mutants were significantly higher than that in WT, and the highest one is *gli1* mutant (Figure 5F), suggesting that the basal G3P levels may not be associated with the alteration of H_2_O_2_ levels in these plants (Figure 3B). The H_2_O_2_ levels in wild-type, *gpdhc1* and *fad-gpdh* were significantly increased under glycerol treatment, while the H_2_O_2_ level in *gli1* was not increased (Figure 5F). In the *FAD-GPDH* ^OE^ lines, the additional G3P was consumed, maintaining cellular redox homeostasis and a normal H_2_O_2_ level under glycerol treatment. Therefore, the ROS level could not be ruled out as a factor in the effects of glycerol on root growth. Interestingly, the PR length was similarly reduced in *FAD-GPDH* ^OE^ lines and WT upon exposure to exogenous H_2_O_2_ (Figure 5G). Further studies are required to determine how redox homeostasis regulated by the G3P shuttle contributes to root growth and development.

Auxin signaling is hypothesized to affect root growth in response to environmental stimuli such as salt, ethylene, nitric oxide and phosphate [22], [25], [65]. For example, SOS3 mediates LR development by regulating auxin redistribution under salt stress [66]. Ethylene increases IAA transport and the expression of *PIN3* and *PIN7*, thereby inhibiting LR growth [67]. Nitric oxide regulates root meristem growth and reduces PIN1-dependent auxin transport [68]. Low phosphate alters root development by regulating auxin sensitivity via TIR1 [22]. Several lines of evidence in our study support the notion that glycerol-induced variations in the PR length and LR abundance may be due to the modification of auxin distribution. First, we found that the auxin distribution pattern was modified in the meristem in response to glycerol treatment using the DR5 marker line. There was an obvious increase in DR5 in the stele cells in the presence of exogenous glycerol (Figure 6C–E and G). In addition, NPA treatment eliminated DR5 accumulation in the stele cells (Figure 6I) and weakened the effect of glycerol on LR formation (Figure 7C). Second, glycerol treatment reduced *PIN7pro::GUS* staining and the expression of the *PIN7pro::PIN7-GFP* protein (Figure 8A and C). The expression of *PIN1* and *PIN7* under exogenous glycerol treatment was also reduced (Figure 8D). Third, auxin signaling mutants, including *tir1* and *arf7*, responded to glycerol treatment differently than WT (Figure 9B), indicating that root architecture remodeling in response to glycerol might be coordinated by auxin redistribution.

Microscopy analysis confirmed that the size and the number of root meristems were dramatically altered under glycerol treatment (Figure 10), which resulted from a decrease in dividing cells in the meristem. At the seedling stage, the number of meristem cells decreased to the point that they were nearly completely depleted (Figure 10A) under glycerol treatment. Interestingly, *gli1* also exhibited a slight decrease in root meristem size and cell number in the presence of glycerol (Figure 10D and E). As a polyalcohol and osmotic protectant, glycerol may impose osmotic stress on cells [69]; however, the effect of osmotic stress on the meristem was minimal. We found that glycerol application reduced the frequency of cell division in the root meristem as determined by the expression of *CycB1;1pro::GUS* (Figure 10H). However, the QC marker genes WOX5 and QC25 were not significantly altered under glycerol treatment (Figure S8). These data indicate that exogenous glycerol reduces mitotic activity in the root meristem.

In conclusion, our results showed that exogenous glycerol treatment alters root architecture by inhibiting PR growth and altering LR development in Arabidopsis. Genetic and biochemical analyses demonstrated that the modified root architecture was due to glycerol dissimilation and possibly impairment of the G3P shuttle. Furthermore, analyses with mutants and marker genes revealed that auxin distribution and root meristematic activity were modified under glycerol treatment as a result of polar auxin transport inhibition. Our study has thus established a link between glycerol dissimilation, auxin transport and root remodeling. Additionally, we identified several important genes involved in the regulation of root development in response to glycerol stress (Figure 11). A full understanding of the effect of glycerol metabolism on root growth has the potential to contribute to the existing knowledge of genes and mechanistic processes that trigger root remodeling under stress.

## Materials and Methods

### Plant Materials and Growth Conditions

All Arabidopsis lines used in this study were in Columbia background. The Arabidopsis T-DNA insertion mutants *gpdhc1* (SALK_020444), *fad-gpdh/sdp6-3* (SALK_080169), *gli1* (SALK_067205) and *act1* (SALK_069657 and CS200/*act1-1* [55]) were obtained from the Arabidopsis Biological Resource Center (ABRC) and identified using a gene-specific and a T-DNA border primer (Table S3). *tir1* [70], *arf7*, *arf19*, *arf7arf19* [16], *slr* [15], *DR5pro::GUS* [56], *PIN1pro::GUS*, *PIN7pro::GUS* [58], *PIN7pro::PIN7-GFP* [6] and *CYCB1;1pro::GUS* [71] were kindly provided by Dr. Jian Xu.

Arabidopsis seeds were sterilized in 50% commercial bleach and plated on 0.5×Murashige and Skoog (MS) medium with 1% sucrose and 0.8% agar (pH 5.8). Seedlings were grown on vertical plates at 22±2°C with humidity ranging from 50% to 60% under long-day (16 h light period) conditions. For growth observations under culture conditions with or without glycerol, the seeds were usually sown directly on the supplemented media; alternatively, the seeds were germinated and grown for 4–5 days on control media and subsequently transferred to plates containing glycerol or other supplements for further growth. When the seedlings were grown on low levels of phosphate, the KH_2_PO_4_ was replaced with KCl to maintain the potassium ion concentration in the medium. The basic modified medium contained 1.65 g L^−1^ NH_4_NO_3_, 1.9 g L^−1^ KNO_3_, 0.44 g L^−1^ CaCl_2_·2H_2_O, 0.37 g L^−1^ MgSO_4_·7H_2_O, 27.8 mg L^−1^ FeSO_4_·7H_2_O, 37.3 mg L^−1^ Na_2_EDTA·2H_2_O, 0.83 mg L^−1^ KI, 6.2 mg L^−1^ H_3_BO_3_, 22.3 mg L^−1^ MnSO_4_·H_2_O, 8.6 mg L^−1^ ZnSO_4_·7H_2_O, 0.25 mg L^−1^ Na_2_MoO_4_·2H_2_O, 0.025 mg L^−1^ CuSO_4_·5H_2_O and 0.025 mg L^−1^ CoCl_2_·6H_2_O. Glycerol, the IAA transport inhibitor NPA and IAA were purchased from Sigma-Aldrich (St. Louis, MO, USA).

### Vector Construction and Plant Transformation

To prepare the *35Spro::AtFAD-GPDH* plasmid, the coding sequence of *FAD-GPDH* was amplified from Arabidopsis cDNA using the primers HJp45 and HJp46 (Table S3). The PCR product was cloned into pMDC83 [72]. For the *AtFAD-GPDHpro::GUS* promoter fusion construct, a 1.35 kb promoter sequence upstream of the start codon of FAD-GPDH was amplified from WT genomic DNA using the primers HJp55 and HJp56 (Table S3). The PCR product was then cloned into the pBI121 vector. The sequences were verified by sequencing analysis at BGI (Shenzhen, China). The resulting plasmid was introduced into the *Agrobacterium* strain GV3101 and used for transformation of Arabidopsis plants using the floral dip method [73].

### Microscopy and Phenotypic Analyses

For root growth analysis, seedlings at various developmental stages were imaged with a digital camera (Canon Powershot s95). PR length, meristem size and cell number were analyzed using ImageJ software (National Institutes of Health, USA). To obtain Nomarski differential interference contrast (DIC) images, the seedlings were cleared and mounted in a chloral hydrate clearing solution (chloral hydrate:water:glycerol, 8∶2∶1, w/v/v) and imaged using a Nikon-Eclipse80i differential interference contrast microscope equipped with a Nikon-DS-RIL CCD camera with DIC optics. Confocal imaging was performed using a Leica TCS SP2 confocal laser scanning microscope. Propidium iodide (Sigma-Aldrich; 10 µg mL^−1^ in distilled water) was used to stain the cell walls (red signal in Figure 8C).

### Histochemical Analysis of GUS Activity

Histochemical analysis of GUS activity was performed according to a previously described protocol [74]. Stained samples were cleared for 24 h in 70% ethanol to remove the chlorophyll. Nomarski DIC images of the GUS staining were obtained as described above.

### Semi-quantitative RT-PCR and Real-time qRT-PCR Analysis

Total RNA samples were isolated from the roots of seedlings grown for the indicated number of days using Trizol reagent (Invitrogen). The RNA samples were treated with DNAse I (Fermentas MBI), and reverse transcription was performed using the First Strand cDNA Synthesis Kit (Fermentas MBI) according to the manufacturer’s protocol. Information regarding the primers used for semi-quantitative RT-PCR is provided in Table S3. *Actin07* was used as an internal control. For the qRT-PCR analysis, the primers were designed to amplify DNA fragments at an annealing temperature of approximately 60°C using the Integrated DNA Technologies (IDT) DNA real-time PCR primer design tool. The primer sequences were obtained with the SIGnAL Gene iSect Tool (Table S3). Real-time qRT-PCR was performed with 2×Bestar Real-Time PCR Master Mix (DBI Bioscience). The reactions were performed and analyzed on a CFX Manager (Bio-Rad) according to the manufacturer’s instructions. PCR was carried out using the following program: 3 min at 95°C, followed by 45 cycles of denaturation for 20 s at 95°C, annealing for 20 s at 56°C and extension for 30 s at 72°C. All transcript levels were normalized to *UBQ10*. All quantitative PCR reactions were performed with at least three biological samples.

### Measurement of Free IAA Content

WT seedlings were treated with 1 mM glycerol for 6 days. Seedling root tips (50 mg) were used for IAA extraction. The extraction and measurement of IAA were performed with some modifications [75]. Each frozen sample was ground to a fine powder in liquid nitrogen and weighed in a 2-mL tube prior to mixing with 1.2 mL of cold extraction buffer (methanol:water, 80∶20, v/v). The samples were vigorously shaken on a shaking bed for 16 h at 4°C in the dark and subsequently centrifuged at 13,000 rpm for 15 min at 4°C. The supernatant was carefully transferred to a new 2-mL tube, and the pellet was resuspended in 400 µL extraction buffer, shaken for 4 h at 4°C and centrifuged. The supernatant was dried by evaporation under the flow of nitrogen gas for approximately 4 h at room temperature and subsequently dissolved in 300 µL methanol. The two supernatants were combined and syringe filtered using a nylon filter (13 mm diameter, 0.22 µm pore size). The free IAA content was assayed with an external standard. Assays were performed by liquid chromatography-mass spectrometry (LC-MS) with four biological replications.

### Measurement of the Cellular Pi Level

The cellular Pi content was determined using the method described by Ames *et al.* [76]. Briefly, fresh tissue (ca. 50 mg) was frozen and ground into a fine powder in liquid nitrogen. After adding 2 mL of 1% glacial acetic acid, the samples were centrifuged at 8,000 g for 5 min at 4°C, and 100 µL of extract was mixed with 200 µL of water and 700 µL of Pi reaction buffer mixture, which contains solution A (0.42% NH_4_MoO_4_ and 2.86% v/v H_2_SO_4_) and solution B (10% w/v L-ascorbic acid) in a ratio of 6∶1. The reaction was allowed to proceed at 37°C for 60 min, and the Pi concentration was calculated as µmol g^−1^ FW based on the OD values at A_820_ using a Pi standard curve.

### Quantification of H_2_O_2_

WT, *gpdhc1, fad-gpdh, gli1*, OE #16 and OE #22 seedlings (approximately 50 mg FW) were sampled at 5 dpg for H_2_O_2_ measurement. The H_2_O_2_ content was determined according to the production of H_2_TiO_4_ using TiCl_4_ as the substrate [77]. The concentration was expressed as nm g^−1^ FW.

### G3P, DHAP and Glycerol Analysis

G3P content in perchloric acid extracts was measured in a reaction mixture containing 1 M glycine, 0.4 M hydrazine buffer (pH 9.5), 1 mM NAD^+^, and 0.75 unit of GPDH (Sigma) as described by Wei *et al.* [78]. The DHAP content was assayed in reaction buffer (50 mM HEPES-NaOH, 1 mM MgCl_2_ and 10 µM NADH) containing 0.75 units of G3P dehydrogenase (Sigma) [79]. The glycerol content in the plant extracts was measured as described [80]. Twenty-five microliters of 50 mM ATP, 25 µL 20 mM β-NAD^+^ and plant extracts (250 µL aliquots) were added successively to a 700 µL glycerol determination reaction buffer containing 137 mM glycine, 686 mM hydrazine, 1.37 mM MgCl_2_ and 17 kU GPDH. The changes in A_340_ were determined using a spectrophotometer (DU730, Beckman Coulter) at 25°C.

### Fatty Acid Analysis

Fatty acid analysis was carried out by placing 7-day-old seedlings in 2 mL of 3% H_2_SO_4_ in methanol [42]. After 60 min incubation at 90°C, 500 µL of hexane containing 0.01% butylated hydroxytoluene was added. The hexane phase was then transferred to vials for gas chromatography (GC). The samples were analyzed using an Agilent 7890 series gas chromatograph with column (HP-INNOWax 19091N-133; 30 m×250 µm×0.25 µm) and quantified with flame ionization detection.

## Supporting Information

Figure S1 **The effect of exogenous glycerol on PR length at different time points after germination.** Wild-type (Col-0) seedlings were grown on the surface of agar plates containing 0.5×MS medium plus 1% (w/v) sucrose with different concentrations of glycerol for the indicated number of days after germination. The PR lengths at different time points after germination are presented (n = 15).
(TIF)

Figure S2 **The effect of exogenous glycerol on PR length and LR number under dark conditions.** (A) Wild-type seedlings were grown on 0.5×Murashige and Skoog (MS) medium plus 1% (w/v) sucrose for 3 days post-germination and subsequently transferred to 0.5×MS media containing 0, 250 µM, 1 mM and 5 mM glycerol for 10 days of growth in dark conditions. Black dots indicated the starting growth positions of the PR tip after shift. (B) The root extension length and (C) lateral root number per plant were recorded. Values are presented as the mean ± SE (n = 24). Asterisks indicate significant differences (control versus treatment: *, p<0.05; **, p<0.01) based on Student’s t-test.
(TIF)

Figure S3 **Dihydroxyacetone phosphate (DHAP) and glycerol levels in seedlings under glycerol treatment.** Wild-type, *gli1*, OE #16 and OE #22 seedlings were grown on agar plates containing 0.5×Murashige and Skoog (MS) medium plus 1% (w/v) sucrose in the absence or presence of 1 mM glycerol for examining glycerol and dihydroxyacetone phosphate (DHAP) levels at the indicated days post-germination (dpg). (A) Glycerol levels of the wild-type seedlings were analyzed from 1–5 dpg and (B) glycerol levels in *gli1*, OE #16, OE #22 and wild-type seedlings at 4 dpg were assayed. The data are presented as the mean ± SE (n = 3–4). (C) DHAP levels of the wild-type seedlings from 1–5 dpg were analyzed. (D) DHAP levels in *gli1*, OE #16, OE #22 and wild-type seedlings at 4 dpg were assayed. The values are expressed as the mean ± SE (n = 4). Different symbols indicate that the means differ significantly by Student’s t-test (*: control versus 1 mM glycerol; #: WT versus mutants or OE lines. *, #: p<0.05; **, ##: p<0.01).
(TIF)

Figure S4 **Exogenous glycerol effects on primary root (PR) length in glycerol-3-phosphate dehydrogenase (GPDH) overexpression lines.** 35S:*BnGPDHp_1_* (A), 35S:*BnGPDHc_1_* (B), 35S:*AtGLY1* (C) and 35S:*GPDHc_2_* (At3g07690) (D) seedlings were grown on the surface of agar plates containing 0.5×Murashige and Skoog (MS) medium for 4 days and subsequently transferred to medium with or without 1 mM glycerol for an additional 3 days. The root lengths were then recorded. Relative PR lengths (%) are shown (A–D), and the values represent the mean ± SE (n >9). Asterisks indicate significant differences (control versus 1 mM glycerol: p<0.05 [*], p<0.01 [**]) by Student’s t-test. The coding sequences of *BnGPDHp_1_* and *BnGPDHc_1_* were amplified from *Brassica napus* cDNA; these genes show high identity with their corresponding genes *AtGPDHp_1_* (At5g40610) and *AtGPDHc_1_* (At2g41540) in Arabidopsis.
(TIF)

Figure S5 **The effect of exogenous glycerol-3-phosphate (G3P) on primary root (PR) length.** (A) Arabidopsis wild-type (Col-0), *gli1*, *gpdhc1*, *fad-gpdh*, OE #16 and OE #22 seedlings grown on media containing 0, 1 mM MgCl_2_ or 1 mM G3P for 5 days are shown. Bar = 0.5 cm. (B) The PR lengths of the seedlings were recorded. The values shown are the means of at least 20 seedlings for each genotype. Asterisks indicate significant differences (*: control versus 1 mM glycerol; #: WT versus mutants or OE lines. *, #: p<0.05; **, ##: p<0.01) by Student’s t-test.
(TIF)

Figure S6 **Expression pattern of the Arabidopsis** ***FAD-GPDH*** **gene.** The gene expression pattern was obtained from the Arabidopsis e-FP Browser website (http://bar.utoronto.ca/efp/cgi-bin/efpWeb.cgi). The figure shows the relative *FAD-GPDH* expression in the root meristem and columella cells.
(TIF)

Figure S7 **Fatty acid levels and root growth of** ***act1*** **mutant and wild-type under glycerol treatment.** (A) The fatty acid levels in 7-day-old wild-type and the *act1* mutants (Salk_069657 and CS200) seedlings are examined. The plants were grown on 0.5×Murashige and Skoog (MS) medium supplemented with 1% (w/v) sucrose with or without 1 mM glycerol. The values shown are the means ± SE (n = 3–4). Asterisks indicate significant differences by Student’s t-test (control versus 1 mM glycerol: #, p<0.05; ##, p<0.01). (B) Wild-type and *act1* mutants (Salk_069657 and CS200) were grown on 0.5×MS medium containing 1% sucrose with or without 1 mM glycerol. The PR lengths of the seedlings were recorded from 2–5 days post-germination (dpg). The values shown are the means of at least 20 seedlings for each genotype. Asterisks indicate significant differences by Student’s t-test (control versus 1 mM glycerol: *, p<0.05: **, p<0.01).
(TIF)

Figure S8 **Glycerol treatment did not affect WOX5-GUS or QC25-GUS staining.** Plants expressing *WOX5pro::GUS* and *QC25pro::GUS* were grown on 0.5×Murashige and Skoog (MS) medium plus 1% (w/v) sucrose media with or without 1 mM glycerol for 6 days and subjected to β-glucuronidase (GUS) staining. Bar = 10 µm.
(TIF)

Table S1 **Effects of glycerol on the number of second-order LRP in plants.** Wild-type seedlings were grown on the surface of agar plates containing 0.5× Murashige and Skoog (MS) medium and 1 mM glycerol for the indicated number of days. The numbers of second-order LRP per plant are presented as the means of 30 seedlings ± SE.
(DOC)

Table S2 **Effects of glycerol on PR length and LR number per plant in 0.1% sucrose.** Wild-type seedlings were grown on the surface of agar plates containing 0.5× Murashige and Skoog (MS) medium with 0.1% sucrose in the presence of different concentrations of glycerol for 7 days. The PR length and the LR number per plant were determined. The values shown represent the means of 12 seedlings ± SE.
(DOC)

Table S3 Primers used in this study.
(DOC)

## References

[1] Petricka JJ, Winter CM, Benfey PN (2012) Control of *Arabidopsis* Root Development. Annu Rev Plant Biol 63: 563–590. 22404466 10.1146/annurev-arplant-042811-105501 PMC3646660

[2] Dhonukshe P, Tanaka H, Goh T, Ebine K, Mähönen AP, et al (2008) Generation of cell polarity in plants links endocytosis, auxin distribution and cell fate decisions. Nature 456: 962–966. 18953331 10.1038/nature07409 PMC2692841

[3] Friml J, Vieten A, Sauer M, Weijers D, Schwarz H, et al (2003) Efflux-dependent auxin gradients establish the apical-basal axis of *Arabidopsis* . Nature 426: 147–153. 14614497 10.1038/nature02085

[4] Benková E, Michniewicz M, Sauer M, Teichmann T, Seifertová D, et al (2003) Local, efflux-dependent auxin gradients as a common module for plant organ formation. Cell 115: 591–602. 14651850 10.1016/s0092-8674(03)00924-3

[5] Péret B, De Rybel B, Casimiro I, Benková E, Swarup R, et al (2009) Arabidopsis lateral root development: an emerging story. Trends Plant Sci 14: 399–408. 19559642 10.1016/j.tplants.2009.05.002

[6] Blilou I, Xu J, Wildwater M, Willemsen V, Paponov I, et al (2005) The PIN auxin efflux facilitator network controls growth and patterning in *Arabidopsis* roots. Nature 433: 39–44. 15635403 10.1038/nature03184

[7] Marchant A, Kargul J, May ST, Muller P, Delbarre A, et al (1999) AUX1 regulates root gravitropism in Arabidopsis by facilitating auxin uptake within root apical tissues. EMBO J 18: 2066–2073. 10205161 10.1093/emboj/18.8.2066 PMC1171291

[8] Terasaka K, Blakeslee JJ, Titapiwatanakun B, Peer WA, Bandyopadhyay A, et al (2005) PGP4, an ATP binding cassette P-glycoprotein, catalyzes auxin transport in *Arabidopsis thaliana* roots. Plant Cell 17: 2922–2939. 16243904 10.1105/tpc.105.035816 PMC1276020

[9] Geldner N, Richter S, Vieten A, Marquardt S, Torres-Ruiz RA, et al (2004) Partial loss-of-function alleles reveal a role for GNOM in auxin transport-related, post-embryonic development of Arabidopsis. Development 131: 389–400. 14681187 10.1242/dev.00926

[10] Sukumar P, LeguÉ V, VayssiÈres A, Martin F, Tuskan GA, et al (2012) Involvement of auxin pathways in modulating root architecture during beneficial plant-microorganism interactions. Plant Cell Environ 36: 909–919. 23145472 10.1111/pce.12036

[11] Malamy JE, Benfey PN (1997) Organization and cell differentiation in lateral roots of *Arabidopsis thaliana* . Development 124: 33–44. 9006065 10.1242/dev.124.1.33

[12] Dubrovsky JG, Sauer M, Napsucialy-Mendivil S, Ivanchenko MG, Friml J, et al (2008) Auxin acts as a local morphogenetic trigger to specify lateral root founder cells. Proc Natl Acad Sci USA 105: 8790–8794. 18559858 10.1073/pnas.0712307105 PMC2438385

[13] Dharmasiri N, Dharmasiri S, Estelle M (2005) The F-box protein TIR1 is an auxin receptor. Nature 435: 441–445. 15917797 10.1038/nature03543

[14] Kepinski S, Leyser O (2005) The Arabidopsis F-box protein TIR1 is an auxin receptor. Nature 435: 446–451. 15917798 10.1038/nature03542

[15] Fukaki H, Tameda S, Masuda H, Tasaka M (2002) Lateral root formation is blocked by a gain-of-function mutation in the SOLITARY-ROOT/IAA14 gene of Arabidopsis. Plant J 29: 153–168. 11862947 10.1046/j.0960-7412.2001.01201.x

[16] Okushima Y, Overvoorde PJ, Arima K, Alonso JM, Chan A, et al (2005) Functional genomic analysis of the AUXIN RESPONSE FACTOR gene family members in *Arabidopsis thaliana*: unique and overlapping functions of ARF7 and ARF19. Plant Cell 17: 444–463. 15659631 10.1105/tpc.104.028316 PMC548818

[17] Dunand C, Crèvecoeur M, Penel C (2007) Distribution of superoxide and hydrogen peroxide in Arabidopsis root and their influence on root development: possible interaction with peroxidases. New Phytol 174: 332–341. 17388896 10.1111/j.1469-8137.2007.01995.x

[18] Gibson SW, Conway AJ, Zheng Z, Uchacz TM, Taylor JL, et al (2012) *Brassica carinata* CIL1 mediates extracellular ROS production during auxin-and ABA-regulated lateral root development. J Plant Biol 55: 361–372.

[19] Lariguet P, Ranocha P, De Meyer M, Barbier O, Penel C, et al (2013) Identification of a hydrogen peroxide signalling pathway in the control of light-dependent germination in Arabidopsis. Planta 238: 381–395. 23716184 10.1007/s00425-013-1901-5

[20] Foreman J, Demidchik V, Bothwell JHF, Mylona P, Miedema H, et al (2003) Reactive oxygen species produced by NADPH oxidase regulate plant cell growth. Nature 422: 442–446. 12660786 10.1038/nature01485

[21] Tsukagoshi H, Busch W, Benfey PN (2010) Transcriptional regulation of ROS controls transition from proliferation to differentiation in the root. Cell 143: 606–616. 21074051 10.1016/j.cell.2010.10.020

[22] Pérez-Torres C-A, López-Bucio J, Cruz-Ramírez A, Ibarra-Laclette E, Dharmasiri S, et al (2008) Phosphate availability alters lateral root development in Arabidopsis by modulating auxin sensitivity via a mechanism involving the TIR1 auxin receptor. Plant Cell 20: 3258–3272. 19106375 10.1105/tpc.108.058719 PMC2630440

[23] Mishra BS, Singh M, Aggrawal P, Laxmi A (2009) Glucose and auxin signaling interaction in controlling *Arabidopsis thaliana* seedlings root growth and development. PLoS One 4: e4502. 19223973 10.1371/journal.pone.0004502 PMC2637607

[24] Lima JE, Kojima S, Takahashi H, Wirén NV (2010) Ammonium triggers lateral root branching in Arabidopsis in an AMMONIUM TRANSPORTER1; 3-dependent manner. Plant Cell 22: 3621–3633. 21119058 10.1105/tpc.110.076216 PMC3015122

[25] Li B, Li Q, Su Y, Chen HAO, Xiong L, et al (2011) Shoot-supplied ammonium targets the root auxin influx carrier AUX1 and inhibits lateral root emergence in Arabidopsis. Plant Cell Environ 34: 933–946. 21342208 10.1111/j.1365-3040.2011.02295.x

[26] Jain A, Poling MD, Karthikeyan AS, Blakeslee JJ, Peer WA, et al (2007) Differential effects of sucrose and auxin on localized phosphate deficiency-induced modulation of different traits of root system architecture in Arabidopsis. Plant Physiol 144: 232–247. 17369438 10.1104/pp.106.092130 PMC1913769

[27] Chitlaru E, Pick U (1991) Regulation of glycerol synthesis in response to osmotic changes in *Dunaliella* . Plant Physiol 96: 50–60. 16668185 10.1104/pp.96.1.50 PMC1080712

[28] Nissen TL, Hamann CW, Kielland-Brandt MC, Nielsen J, Villadsen J (2000) Anaerobic and aerobic batch cultivations of *Saccharomyces cerevisiae* mutants impaired in glycerol synthesis. Yeast 16: 463–474. 10705374 10.1002/(SICI)1097-0061(20000330)16:5<463::AID-YEA535>3.0.CO;2-3

[29] Lin ECC (1976) Glycerol dissimilation and its regulation in bacteria. Annu Rev Microbiol 30: 535–578. 825019 10.1146/annurev.mi.30.100176.002535

[30] Ben-Amotz A, Avron M (1973) The role of glycerol in the osmotic regulation of the halophilic alga *Dunaliella parva* . Plant Physiol 51: 875–878. 16658431 10.1104/pp.51.5.875 PMC366367

[31] Zhuge J, Fang HY, Wang ZX, Chen DZ, Jin HR, et al (2001) Glycerol production by a novel osmotolerant yeast *Candida glycerinogenes* . Appl Microbiol Biot 55: 686–692. 10.1007/s002530100596 11525615

[32] Gerber DW, Byerrum RU, Gee RW, Tolbert NE (1988) Glycerol concentrations in crop plants. Plant Sci 56: 31–38.

[33] Eastmond PJ (2004) Glycerol-insensitive Arabidopsis mutants: *gli1* seedlings lack glycerol kinase, accumulate glycerol and are more resistant to abiotic stress. Plant J 37: 617–625. 14756771 10.1111/j.1365-313x.2003.01989.x

[34] Leegood RC, Labate CA, Huber SC, Neuhaus HE, Stitt M (1988) Phosphate sequestration by glycerol and its effects on photosynthetic carbon assimilation by leaves. Planta 176: 117–126. 24220742 10.1007/BF00392487

[35] Aubert S, Gout E, Bligny R, Douce R (1994) Multiple effects of glycerol on plant cell metabolism. Phosphorus-31 nuclear magnetic resonance studies. J Biol Chem 269: 21420–21427. 8063774

[36] Vigeolas H, Geigenberger P (2004) Increased levels of glycerol-3-phosphate lead to a stimulation of flux into triacylglycerol synthesis after supplying glycerol to developing seeds of *Brassica napus L*. in planta. Planta 219: 827–835. 15107995 10.1007/s00425-004-1273-y

[37] Kachroo A, Venugopal SC, Lapchyk L, Falcone D, Hildebrand D, et al (2004) Oleic acid levels regulated by glycerolipid metabolism modulate defense gene expression in Arabidopsis. Proc Natl Acad Sci USA 101: 5152–5157. 15044700 10.1073/pnas.0401315101 PMC387389

[38] Kachroo P, Venugopal SC, Navarre DA, Lapchyk L, Kachroo A (2005) Role of salicylic acid and fatty acid desaturation pathways in ssi2-mediated signaling. Plant Physiol 139: 1717–1735. 16306139 10.1104/pp.105.071662 PMC1310554

[39] Bellettre A, Couillerot J-P, Blervacq A-S, Aubert S, Gout E, et al (2001) Glycerol effects both carbohydrate metabolism and cytoskeletal rearrangements during the induction of somatic embryogenesis in chicory leaf tissues. Plant Physiol Biochem 39: 503–511.

[40] Inoue Y, Moriyasu Y (2006) Autophagy is not a main contributor to the degradation of phospholipids in tobacco cells cultured under sucrose starvation conditions. Plant Cell Physiol 47: 471–480. 16449232 10.1093/pcp/pcj013

[41] Lin ECC (1977) Glycerol utilization and its regulation in mammals. Annu Rev Biochem 46: 765–795. 197882 10.1146/annurev.bi.46.070177.004001

[42] Chanda B, Venugopal SC, Kulshrestha S, Navarre DA, Downie B, et al (2008) Glycerol-3-Phosphate levels are associated with basal resistance to the hemibiotrophic fungus *Colletotrichum higginsianum* in Arabidopsis. Plant Physiol 147: 2017–2029. 18567828 10.1104/pp.108.121335 PMC2492641

[43] Chanda B, Xia Y, Mandal MK, Yu K, Sekine K-T, et al (2011) Glycerol-3-phosphate is a critical mobile inducer of systemic immunity in plants. Nat Genet 43: 421–427. 21441932 10.1038/ng.798

[44] Quettier A-L, Shaw E, Eastmond PJ (2008) *SUGAR-DEPENDENT6* encodes a mitochondrial flavin adenine dinucleotide-dependent Glycerol-3-P dehydrogenase, which is required for glycerol catabolism and postgerminative seedling growth in Arabidopsis. Plant Physiol 148: 519–528. 18599644 10.1104/pp.108.123703 PMC2528096

[45] Shen W, Wei Y, Dauk M, Tan Y, Taylor DC, et al (2006) Involvement of a glycerol-3-phosphate dehydrogenase in modulating the NADH/NAD^+^ ratio provides evidence of a mitochondrial Glycerol-3-Phosphate shuttle in Arabidopsis. Plant Cell 18: 422–441. 16415206 10.1105/tpc.105.039750 PMC1356549

[46] Vigeolas H, Waldeck P, Zank T, Geigenberger P (2007) Increasing seed oil content in oil-seed rape (*Brassica napus L.*) by over-expression of a yeast glycerol-3-phosphate dehydrogenase under the control of a seed-specific promoter. Plant Biotechnol J 5: 431–441. 17430545 10.1111/j.1467-7652.2007.00252.x

[47] Shen W, Li JQ, Dauk M, Huang Y, Periappuram C, et al (2010) Metabolic and transcriptional responses of glycerolipid pathways to a perturbation of Glycerol-3-Phosphate metabolism in Arabidopsis. J Biol Chem 285: 22957–22965. 20304913 10.1074/jbc.M109.097758 PMC2906289

[48] Yang Y, Zhao J, Liu P, Xing H, Li C, et al (2013) Glycerol-3-Phosphate Metabolism in Wheat Contributes to Systemic Acquired Resistance against *Puccinia striiformis f. sp. tritici* . PloS One 8: e81756. 24312351 10.1371/journal.pone.0081756 PMC3843702

[49] Yu K, Soares JM, Mandal MK, Wang C, Chanda B, et al (2013) A Feedback Regulatory Loop between G3P and Lipid Transfer Proteins DIR1 and AZI1 Mediates Azelaic-Acid-Induced Systemic Immunity. Cell Reports 3: 1266–1278. 23602565 10.1016/j.celrep.2013.03.030

[50] Zhang H, Jennings A, Barlow PW, Forde BG (1999) Dual pathways for regulation of root branching by nitrate. Proc Natl Acad Sci USA 96: 6529–6534. 10339622 10.1073/pnas.96.11.6529 PMC26916

[51] Shen W, Wei Y, Dauk M, Zheng Z, Zou J (2003) Identification of a mitochondrial glycerol-3-phosphate dehydrogenase from *Arabidopsis thaliana*: evidence for a mitochondrial glycerol-3-phosphate shuttle in plants. FEBS Lett 536: 92–96. 12586344 10.1016/s0014-5793(03)00033-4

[52] Williamson LC, Ribrioux SPCP, Fitter AH, Leyser HMO (2001) Phosphate availability regulates root system architecture in Arabidopsis. Plant Physiol 126: 875–882. 11402214 10.1104/pp.126.2.875 PMC111176

[53] Mandal MK, Chandra-Shekara A, Jeong R-D, Yu K, Zhu S, et al (2012) Oleic Acid–Dependent Modulation of NITRIC OXIDE ASSOCIATED1 Protein Levels Regulates Nitric Oxide–Mediated Defense Signaling in *Arabidopsis* . Plant Cell 24: 1654–1674. 22492810 10.1105/tpc.112.096768 PMC3398570

[54] Kunst L, Somerville C (1988) Altered regulation of lipid biosynthesis in a mutant of Arabidopsis deficient in chloroplast glycerol-3-phosphate acyltransferase activity. Proc Natl Acad Sci USA 85: 4143–4147. 16593939 10.1073/pnas.85.12.4143 PMC280382

[55] Xu C YB, Cornish AJ, Froehlich JE, Benning C (2006) Phosphatidylglycerol biosynthesis in chloroplasts of Arabidopsis mutants deficient in acyl-ACP glycerol-3-phosphate acyltransferase. Plant J 47: 296–309. 16774646 10.1111/j.1365-313X.2006.02790.x

[56] Ulmasov T, Murfett J, Hagen G, Guilfoyle TJ (1997) Aux/IAA proteins repress expression of reporter genes containing natural and highly active synthetic auxin response elements. Plant Cell 9: 1963–1971. 9401121 10.1105/tpc.9.11.1963 PMC157050

[57] Casimiro I, Marchant A, Bhalerao RP, Beeckman T, Dhooge S, et al (2001) Auxin transport promotes Arabidopsis lateral root initiation. Plant Cell 13: 843–852. 11283340 10.1105/tpc.13.4.843 PMC135543

[58] Vieten A, Vanneste S, Wiśniewska J, Benková E, Benjamins R, et al (2005) Functional redundancy of PIN proteins is accompanied by auxin-dependent cross-regulation of PIN expression. Development 132: 4521–4531. 16192309 10.1242/dev.02027

[59] Hippmann H, Heinz E (1976) Glycerol kinase in leaves. Zeitschrift für Pflanzenphysiologie 79: 408–418.

[60] Elvin C, Hardy C, Rosenberg H (1985) Pi exchange mediated by the GlpT-dependent sn-glycerol-3-phosphate transport system in *Escherichia coli* . J Bacteriol 161: 1054–1058. 3882662 10.1128/jb.161.3.1054-1058.1985 PMC215006

[61] Huang Y, Lemieux MJ, Song J, Auer M, Wang D-N (2003) Structure and mechanism of the glycerol-3-phosphate transporter from *Escherichia coli* . Science 301: 616–620. 12893936 10.1126/science.1087619

[62] Ramaiah M, Jain A, Baldwin JC, Karthikeyan AS, Raghothama KG (2011) Characterization of the phosphate starvation-induced *glycerol-3-phosphate permease* gene family in Arabidopsis. Plant Physiol 157: 279–291. 21788361 10.1104/pp.111.178541 PMC3165876

[63] Caparrós-Martín JA, Reiland S, Köchert K, Cutanda MC, Culiáñez-Macià FA (2007) *Arabidopsis thaliana* AtGpp1 and AtGpp2: two novel low molecular weight phosphatases involved in plant glycerol metabolism. Plant Mol Biol 63: 505–517. 17136424 10.1007/s11103-006-9104-0

[64] Påhlman A-K, Granath K, Ansell R, Hohmann S, Adler L (2001) The yeast glycerol 3-phosphatases Gpp1p and Gpp2p are required for glycerol biosynthesis and differentially involved in the cellular responses to osmotic, anaerobic, and oxidative stress. J Biol Chem 276: 3555–3563. 11058591 10.1074/jbc.M007164200

[65] Giehl RFH, Lima JE, von Wirén N (2012) Localized iron supply triggers lateral root elongation in *Arabidopsis* by altering the AUX1-mediated auxin distribution. Plant Cell 24: 33–49. 22234997 10.1105/tpc.111.092973 PMC3289578

[66] Zhao Y, Wang T, Zhang W, Li X (2011) SOS3 mediates lateral root development under low salt stress through regulation of auxin redistribution and maxima in Arabidopsis. New Phytol 189: 1122–1134. 21087263 10.1111/j.1469-8137.2010.03545.x

[67] Lewis DR, Negi S, Sukumar P, Muday GK (2011) Ethylene inhibits lateral root development, increases IAA transport and expression of PIN3 and PIN7 auxin efflux carriers. Development 138: 3485–3495. 21771812 10.1242/dev.065102

[68] Fernández-Marcos M, Sanz L, Lewis DR, Muday GK, Lorenzo O (2011) Nitric oxide causes root apical meristem defects and growth inhibition while reducing PIN-FORMED 1 (PIN1)-dependent acropetal auxin transport. Proc Natl Acad Sci USA 108: 18506–18511. 22021439 10.1073/pnas.1108644108 PMC3215072

[69] Siderius M, Van Wuytswinkel O, Reijenga KA, Kelders M, Mager WH (2002) The control of intracellular glycerol in *Saccharomyces cerevisiae* influences osmotic stress response and resistance to increased temperature. Mol Microbiol 36: 1381–1390. 10.1046/j.1365-2958.2000.01955.x 10931288

[70] Ruegger M, Dewey E, Gray WM, Hobbie L, Turner J, et al (1998) The TIR1 protein of *Arabidopsis* functions in auxin response and is related to human SKP2 and yeast Grr1p. Genes Dev 12: 198–207. 9436980 10.1101/gad.12.2.198 PMC316440

[71] Colón-Carmona A, You R, Haimovitch-Gal T, Doerner P (2003) Spatio-temporal analysis of mitotic activity with a labile cyclin-GUS fusion protein. Plant J 20: 503–508. 10.1046/j.1365-313x.1999.00620.x 10607302

[72] Curtis MD, Grossniklaus U (2003) A gateway cloning vector set for high-throughput functional analysis of genes in planta. Plant Physiol 133: 462–469. 14555774 10.1104/pp.103.027979 PMC523872

[73] Clough SJ, Bent AF (1998) Floral dip: a simplified method for *Agrobacterium*-mediated transformation of *Arabidopsis thaliana* . Plant J 16: 735–743. 10069079 10.1046/j.1365-313x.1998.00343.x

[74] Xu J, Scheres B (2005) Dissection of Arabidopsis ADP-RIBOSYLATION FACTOR 1 function in epidermal cell polarity. Plant Cell 17: 525–536. 15659621 10.1105/tpc.104.028449 PMC548823

[75] Liu H, Li X, Xiao J, Wang S (2012) A convenient method for simultaneous quantification of multiple phytohormones and metabolites: application in study of rice-bacterium interaction. Plant Methods 8: 2. 22243810 10.1186/1746-4811-8-2 PMC3274484

[76] Ames BN (1966) Assay of inorganic phosphate, total phosphate and phosphatase. Methods Enzymol 8: 115–118.

[77] Min L, Zhu L, Tu L, Deng F, Yuan D, et al (2013) Cotton GhCKI disrupts normal male reproduction by delaying tapetum programmed cell death via inactivating starch synthase. Plant J 75: 823–835. 23662698 10.1111/tpj.12245

[78] Wei Y, Shen W, Dauk M, Wang F, Selvaraj G, et al (2004) Targeted gene disruption of glycerol-3-phosphate dehydrogenase in *Colletotrichum gloeosporioides* reveals evidence that glycerol is a significant transferred nutrient from host plant to fungal pathogen. J Exp Bot 279: 429–435. 10.1074/jbc.M308363200 14563847

[79] Häusler RE, Fischer KL, Flügge U-I (2000) Determination of low-abundant metabolites in plant extracts by NAD(P)H fluorescence with a microtiter plate reader. Anal Biochem 281: 1–8. 10847603 10.1006/abio.2000.4556

[80] Wieland OH (1974) Glycerol assay: UV-method. In: Bergmeyer H-U (ed.), Methods of enzymatic analysis, New York, Academic Press, 504–509.

