# Supplementary figures and images for: Glycerol Affects Root Development through Regulation of Multiple Pathways in Arabidopsis
Source: PLoS One. 2014 Jan 22;9(1):e86269. doi: 10.1371/journal.pone.0086269 (PMC3899222; doi:10.1371/journal.pone.0086269)

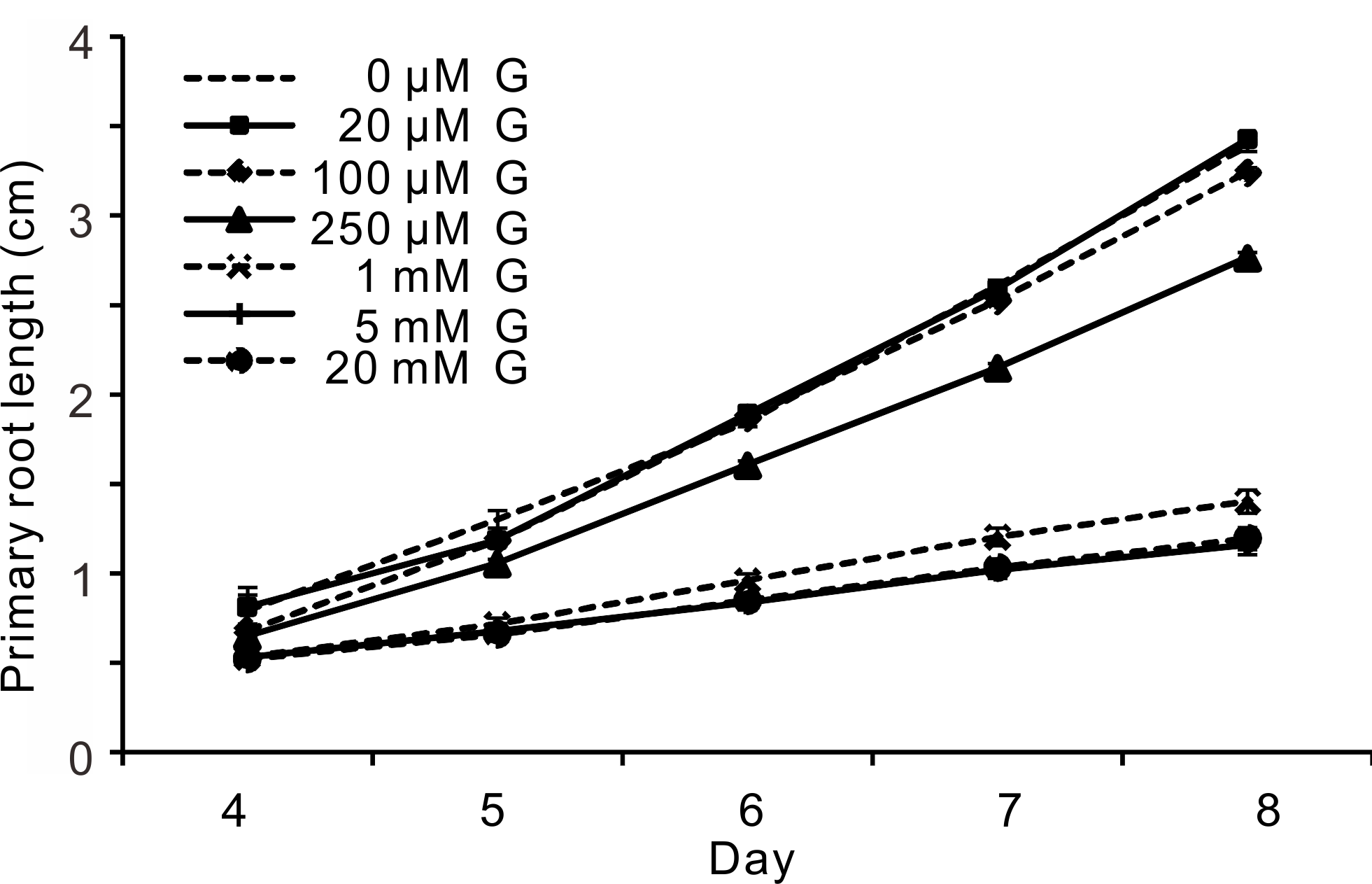

Supplement: Figure S1 — The effect of exogenous glycerol on PR length at different time points after germination. Wild-type (Col-0) seedlings were grown on the surface of agar plates containing 0.5×MS medium plus 1% (w/v) sucrose with different concentrations of glycerol for the indicated number of days after germination. The PR lengths at different time points after germination are presented (n = 15). (TIF) [file pone.0086269.s001.tif]

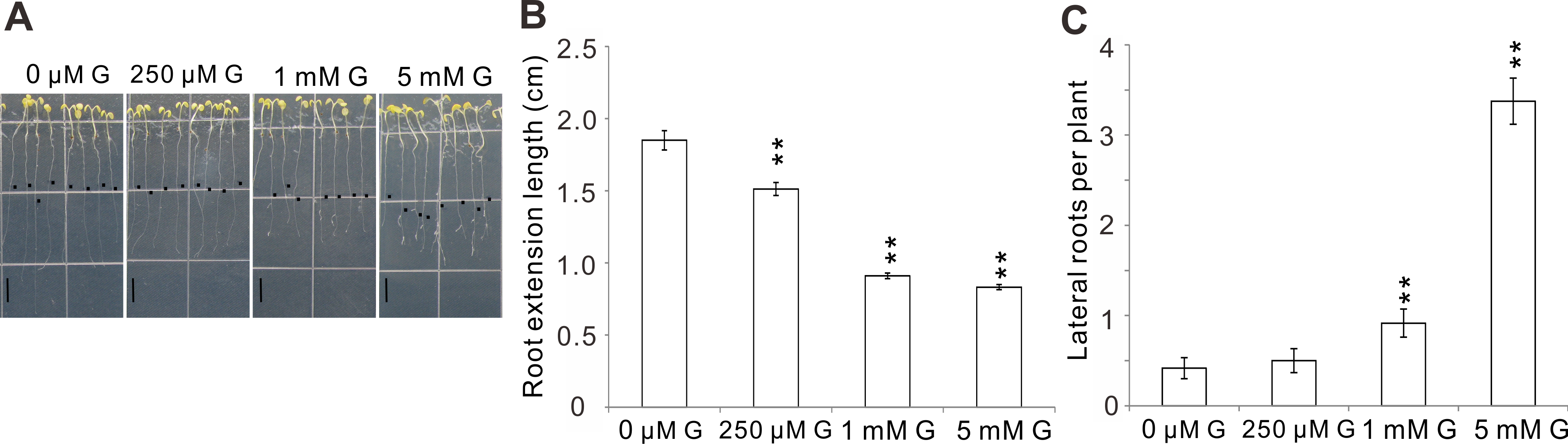

Supplement: Figure S2 — The effect of exogenous glycerol on PR length and LR number under dark conditions. (A) Wild-type seedlings were grown on 0.5×Murashige and Skoog (MS) medium plus 1% (w/v) sucrose for 3 days post-germination and subsequently transferred to 0.5×MS media containing 0, 250 µM, 1 mM and 5 mM glycerol for 10 days of growth in dark conditions. Black dots indicated the starting growth positions of the PR tip after shift. (B) The root extension length and (C) lateral root number per plant were recorded. Values are presented as the mean ± SE (n = 24). Asterisks indicate significant differences (control versus treatment: *, p<0.05; **, p<0.01) based on Student’s t-test. (TIF) [file pone.0086269.s002.tif]

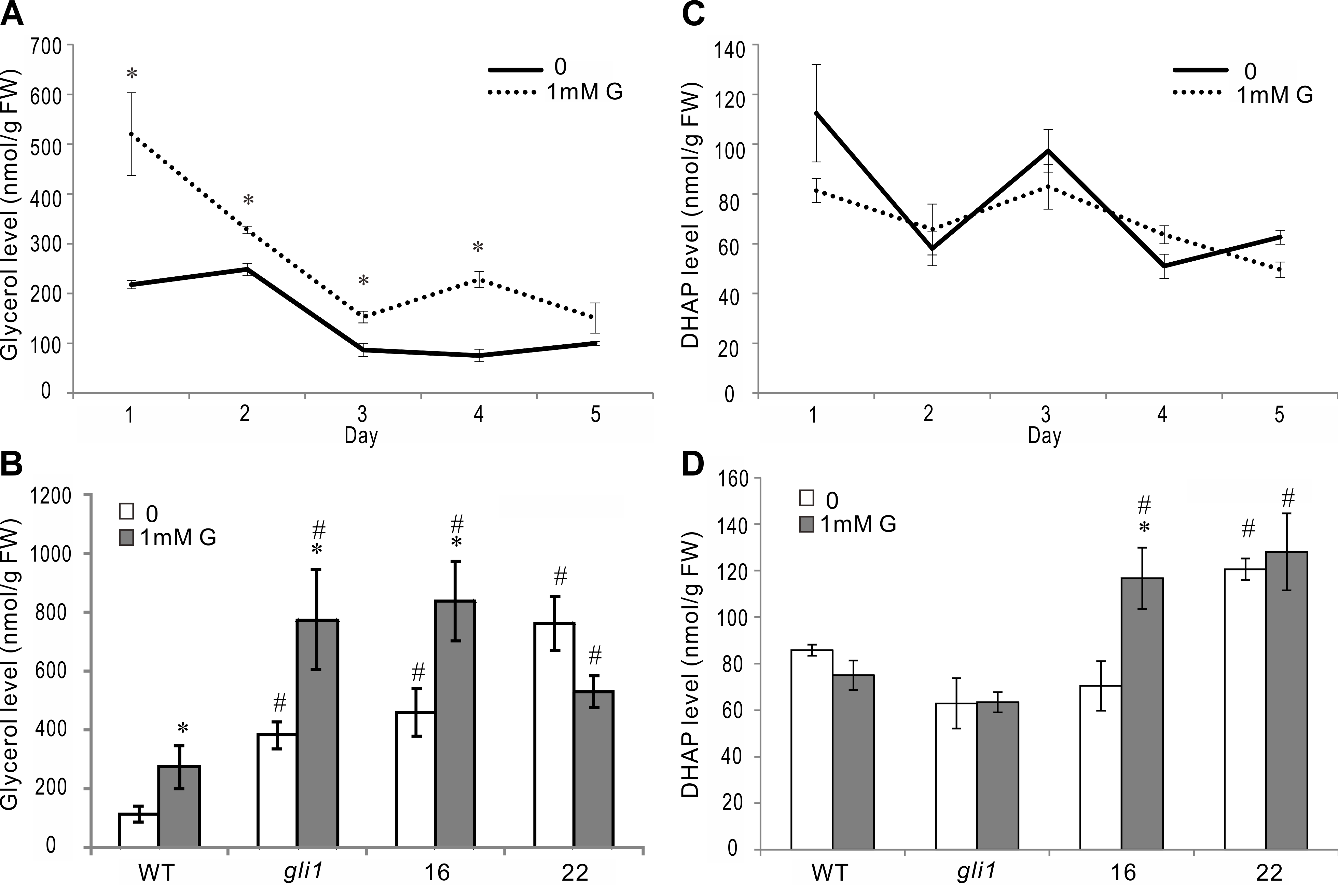

Supplement: Figure S3 — Dihydroxyacetone phosphate (DHAP) and glycerol levels in seedlings under glycerol treatment. Wild-type, gli1, OE #16 and OE #22 seedlings were grown on agar plates containing 0.5×Murashige and Skoog (MS) medium plus 1% (w/v) sucrose in the absence or presence of 1 mM glycerol for examining glycerol and dihydroxyacetone phosphate (DHAP) levels at the indicated days post-germination (dpg). (A) Glycerol levels of the wild-type seedlings were analyzed from 1–5 dpg and (B) glycerol levels in gli1, OE #16, OE #22 and wild-type seedlings at 4 dpg were assayed. The data are presented as the mean ± SE (n = 3–4). (C) DHAP levels of the wild-type seedlings from 1–5 dpg were analyzed. (D) DHAP levels in gli1, OE #16, OE #22 and wild-type seedlings at 4 dpg were assayed. The values are expressed as the mean ± SE (n = 4). Different symbols indicate that the means differ significantly by Student’s t-test (*: control versus 1 mM glycerol; #: WT versus mutants or OE lines. *, #: p<0.05; **, ##: p<0.01). (TIF) [file pone.0086269.s003.tif]

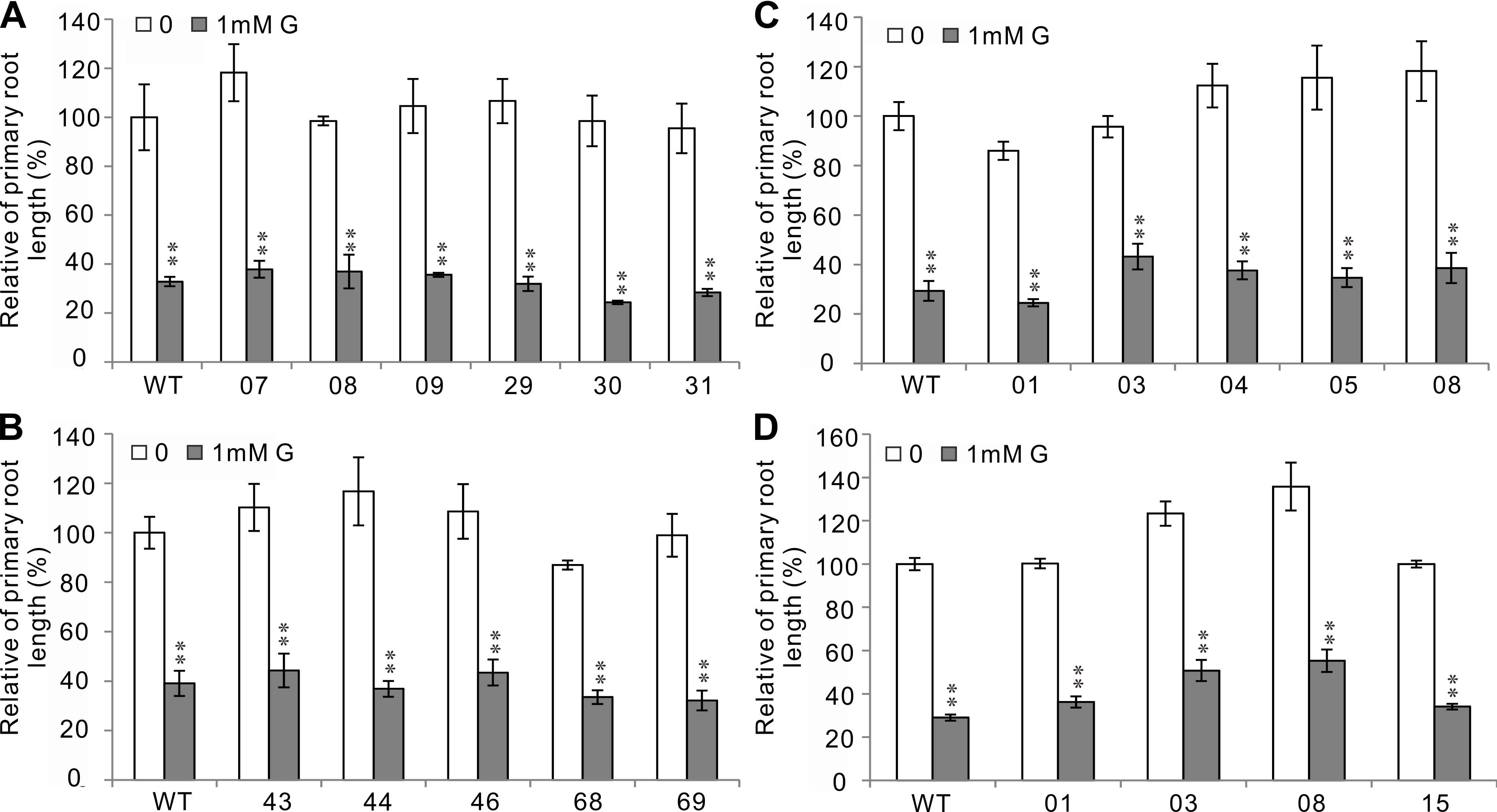

Supplement: Figure S4 — Exogenous glycerol effects on primary root (PR) length in glycerol-3-phosphate dehydrogenase (GPDH) overexpression lines. 35S:BnGPDHp1 (A), 35S:BnGPDHc1 (B), 35S:AtGLY1 (C) and 35S:GPDHc2 (At3g07690) (D) seedlings were grown on the surface of agar plates containing 0.5×Murashige and Skoog (MS) medium for 4 days and subsequently transferred to medium with or without 1 mM glycerol for an additional 3 days. The root lengths were then recorded. Relative PR lengths (%) are shown (A–D), and the values represent the mean ± SE (n >9). Asterisks indicate significant differences (control versus 1 mM glycerol: p<0.05 [*], p<0.01 [**]) by Student’s t-test. The coding sequences of BnGPDHp1 and BnGPDHc1 were amplified from Brassica napus cDNA; these genes show high identity with their corresponding genes AtGPDHp1 (At5g40610) and AtGPDHc1 (At2g41540) in Arabidopsis. (TIF) [file pone.0086269.s004.tif]

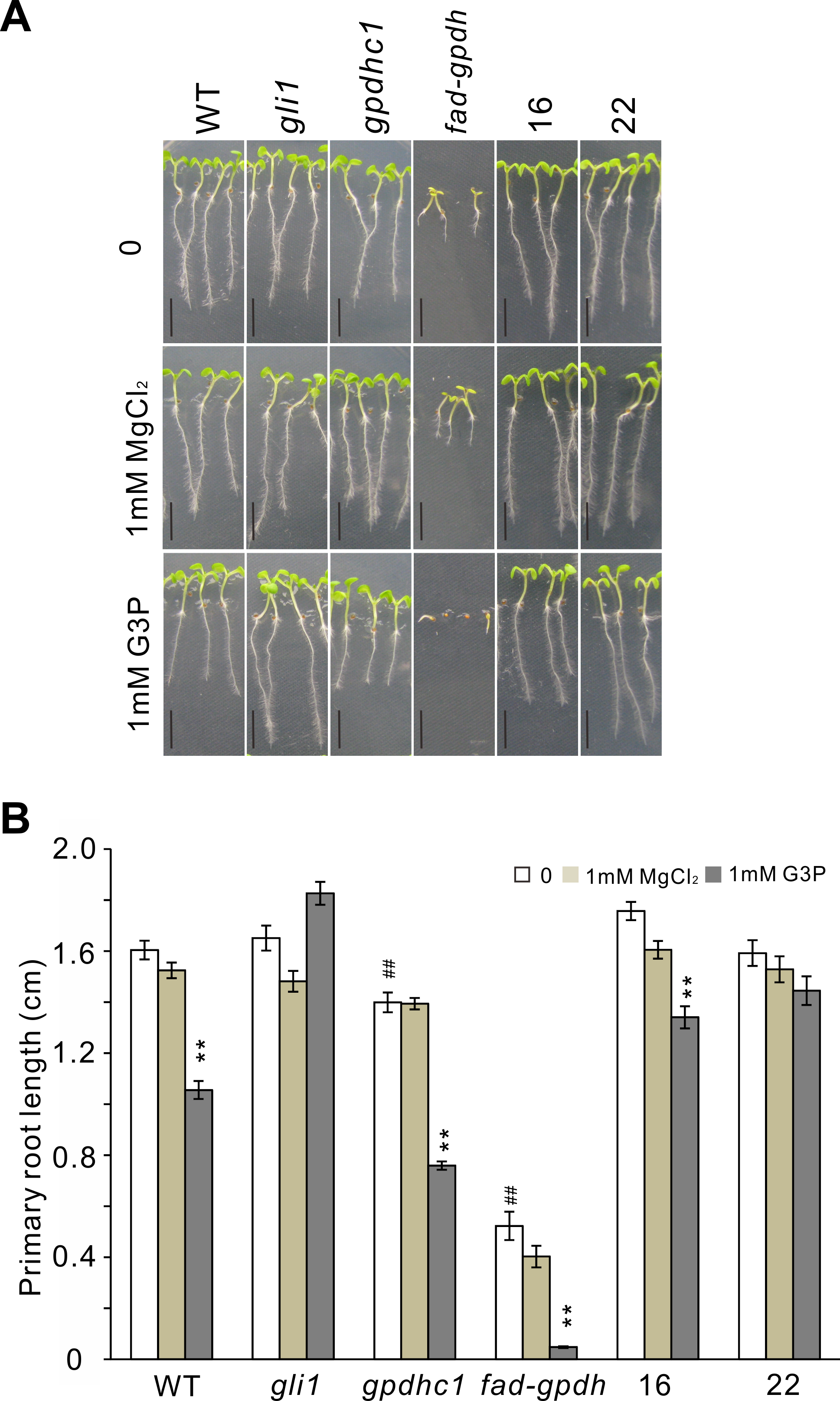

Supplement: Figure S5 — The effect of exogenous glycerol-3-phosphate (G3P) on primary root (PR) length. (A) Arabidopsis wild-type (Col-0), gli1, gpdhc1, fad-gpdh, OE #16 and OE #22 seedlings grown on media containing 0, 1 mM MgCl2 or 1 mM G3P for 5 days are shown. Bar = 0.5 cm. (B) The PR lengths of the seedlings were recorded. The values shown are the means of at least 20 seedlings for each genotype. Asterisks indicate significant differences (*: control versus 1 mM glycerol; #: WT versus mutants or OE lines. *, #: p<0.05; **, ##: p<0.01) by Student’s t-test. (TIF) [file pone.0086269.s005.tif]

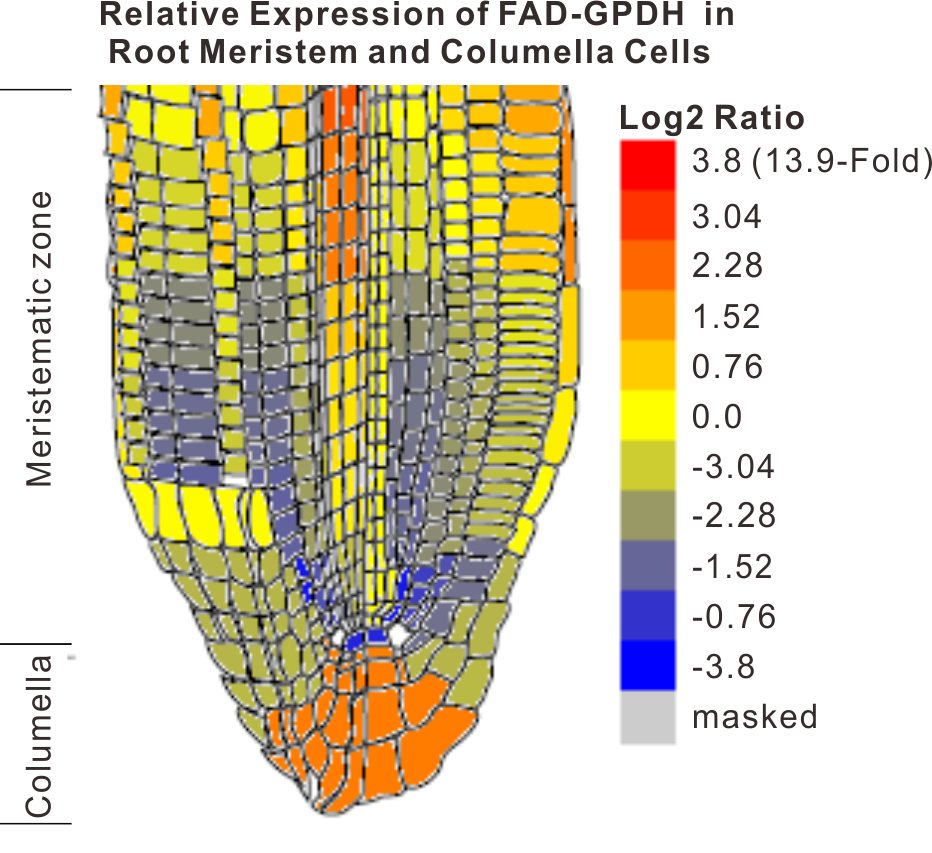

Supplement: Figure S6 — Expression pattern of the Arabidopsis FAD-GPDH gene. The gene expression pattern was obtained from the Arabidopsis e-FP Browser website (http://bar.utoronto.ca/efp/cgi-bin/efpWeb.cgi). The figure shows the relative FAD-GPDH expression in the root meristem and columella cells. (TIF) [file pone.0086269.s006.tif]

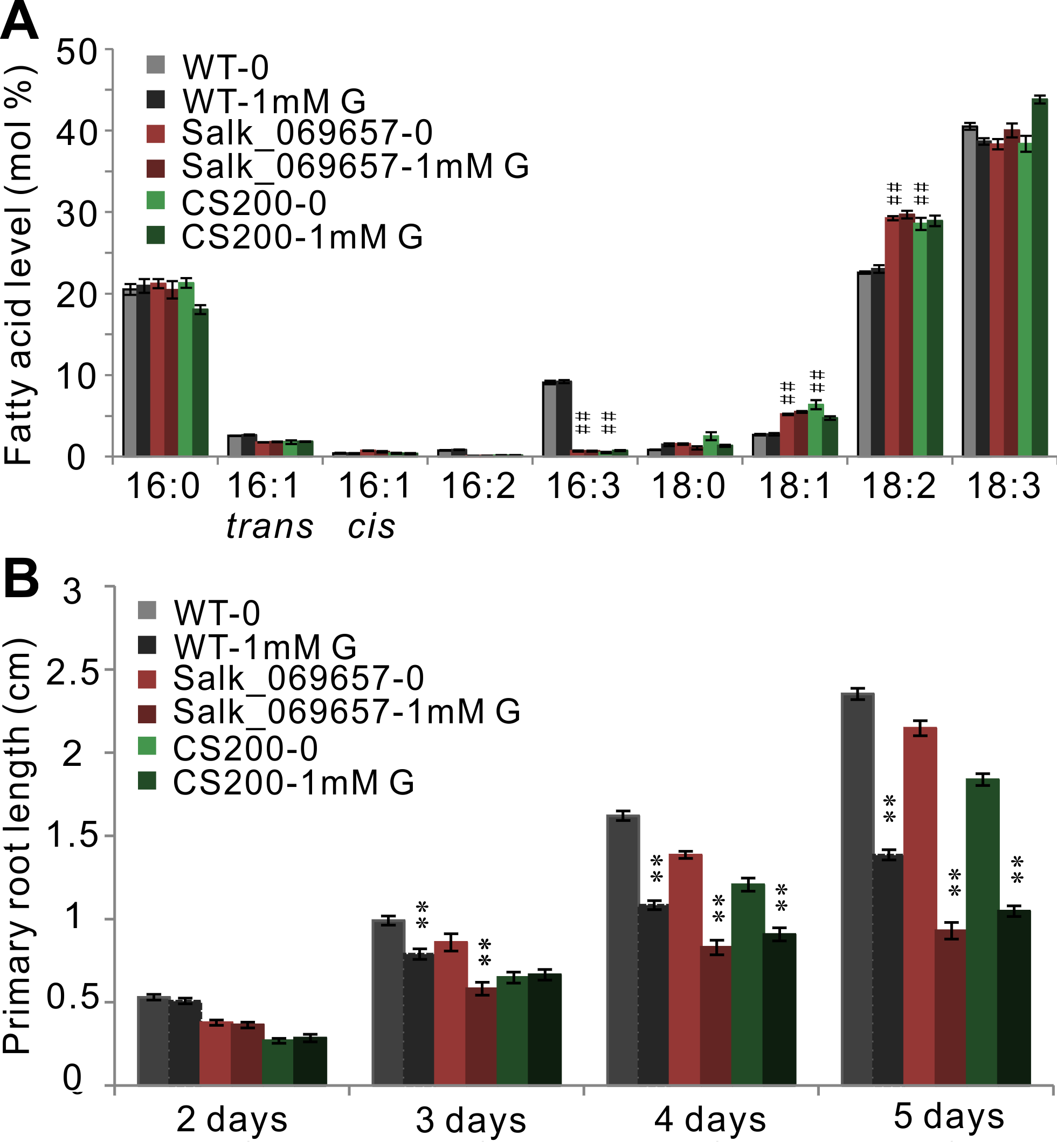

Supplement: Figure S7 — Fatty acid levels and root growth of act1 mutant and wild-type under glycerol treatment. (A) The fatty acid levels in 7-day-old wild-type and the act1 mutants (Salk_069657 and CS200) seedlings are examined. The plants were grown on 0.5×Murashige and Skoog (MS) medium supplemented with 1% (w/v) sucrose with or without 1 mM glycerol. The values shown are the means ± SE (n = 3–4). Asterisks indicate significant differences by Student’s t-test (control versus 1 mM glycerol: #, p<0.05; ##, p<0.01). (B) Wild-type and act1 mutants (Salk_069657 and CS200) were grown on 0.5×MS medium containing 1% sucrose with or without 1 mM glycerol. The PR lengths of the seedlings were recorded from 2–5 days post-germination (dpg). The values shown are the means of at least 20 seedlings for each genotype. Asterisks indicate significant differences by Student’s t-test (control versus 1 mM glycerol: *, p<0.05: **, p<0.01). (TIF) [file pone.0086269.s007.tif]

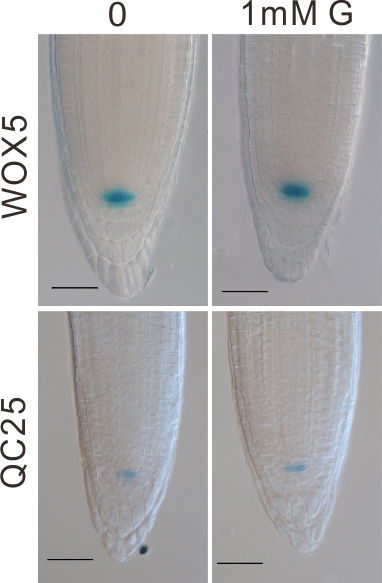

Supplement: Figure S8 — Glycerol treatment did not affect WOX5-GUS or QC25-GUS staining. Plants expressing WOX5pro::GUS and QC25pro::GUS were grown on 0.5×Murashige and Skoog (MS) medium plus 1% (w/v) sucrose media with or without 1 mM glycerol for 6 days and subjected to β-glucuronidase (GUS) staining. Bar = 10 µm. (TIF) [file pone.0086269.s008.tif]
