# Supplementary material for: Glycerol Affects Root Development through Regulation of Multiple Pathways in Arabidopsis
Source: PLoS One. 2014 Jan 22;9(1):e86269. doi: 10.1371/journal.pone.0086269 (PMC3899222; doi:10.1371/journal.pone.0086269)
Supplement: Table S1 — Effects of glycerol on the number of second-order LRP in plants. Wild-type seedlings were grown on the surface of agar plates containing 0.5× Murashige and Skoog (MS) medium and 1 mM glycerol for the indicated number of days. The numbers of second-order LRP per plant are presented as the means of 30 seedlings ± SE. (DOC) [file pone.0086269.s009.doc]

**Table S1.** **Effects of glycerol on the number of second-order LRP in plants.**

|  | 2 days | 4 days | 6 days | | | 8 days | | | |
| --- | --- | --- | --- | --- | --- | --- | --- | --- | --- |
|  |  |  | 2A | 2B | 2C | 2A | 2B | 2C | 2D |
| 0 | 0 | 0 | 0.48 ± 0.13 | 0.07 ± 0.05 | 0 | 1.30 ± 0.23 | 0.70 ± 0.27 | 0.39 ± 0.12 | 0.06 ± 0.06 |
| 1 mM glycerol | 0 | 0 | 2.08 ± 0.23 | 0.52 ± 0.13 | 0.12 ± 0.08 | 3.19 ± 0.43 | 2.43 ± 0.43 | 1.46 ± 0.25 | 0.62 ± 0.14 |

Table S1. Effects of glycerol on the number of second-order LRP in plants. Wild-type seedlings were grown on the surface of agar plates containing 0.5x Murashige and Skoog (MS) medium and 1 mM glycerol for the indicated number of days. The numbers of second-order LRP per plant are presented as the means of 30 seedlings ± SE.
