# Supplementary material for: Glycerol Affects Root Development through Regulation of Multiple Pathways in Arabidopsis
Source: PLoS One. 2014 Jan 22;9(1):e86269. doi: 10.1371/journal.pone.0086269 (PMC3899222; doi:10.1371/journal.pone.0086269)
Supplement: Table S2 — Effects of glycerol on PR length and LR number per plant in 0.1% sucrose. Wild-type seedlings were grown on the surface of agar plates containing 0.5× Murashige and Skoog (MS) medium with 0.1% sucrose in the presence of different concentrations of glycerol for 7 days. The PR length and the LR number per plant were determined. The values shown represent the means of 12 seedlings ± SE. (DOC) [file pone.0086269.s010.doc]

**Table S2.** **Effects of glycerol on PR length and LR number per plant in 0.1% sucrose.**

|  | 0 | 250 μM glycerol | 1 mM glycerol | 5 mM glycerol |
| --- | --- | --- | --- | --- |
| PR length (cm) | 1.34 ± 0.09 | 1.39 ± 0.09 | 0.61 ± 0.03 | 0.54 ± 0.02 |
| LR number per plant | 0 | 0 | 1.78 ± 0.21 | 3.66 ± 0.38 |

Table S2. Effects of glycerol on PR length and LR number per plant in 0.1% sucrose. Wild-type seedlings were grown on the surface of agar plates containing 0.5x Murashige and Skoog (MS) medium with 0.1% sucrose in the presence of different concentrations of glycerol for 7 days. The PR length and the LR number per plant were determined. The values shown represent the means of 12 seedlings ± SE.
