# Supplementary material for: Glycerol Affects Root Development through Regulation of Multiple Pathways in Arabidopsis
Source: PLoS One. 2014 Jan 22;9(1):e86269. doi: 10.1371/journal.pone.0086269 (PMC3899222; doi:10.1371/journal.pone.0086269)
Supplement: Table S3 — Primers used in this study. (DOC) [file pone.0086269.s011.doc]

**Table S3.** Primers used in this study

| *FAD-GPDH* overexpression construct | |
| --- | --- |
| HJp45 | Forward: AGttaattaaATGTCTCTCGCTTCCATTC |
| HJp46 | Reverse: CTggcgcgccTTAGTTGTGCTTGCCATC |
| *FAD-GPDH p*romoter construct | |
| HJp55 | Forward: AGAGaagcttAATTCGTGTCGGTGTTAC |
| HJp56 | Reverse: AGCTtctagaGGAATTTAGGAGAGATTT |
| Primers used to identify the knockout mutants | |
| *gpdhc1* | Forward: ATGGTGGGAAGCATTGAG |
|  | Reverse: TCAAGGCTGACCAAGAAG |
|  | T-DNA border: TCCAAACTGGAACAACACTCAA |
| *gli1* | Forward: GATTTGGCAGCAATGGTA |
|  | Reverse: TTAGATAGAGAGGTCAGCG |
|  | T-DNA border: TCCAAACTGGAACAACACTCAA |
| *fad-gpdh* | Forward: TTGGGAAGAACAGTAGC |
|  | Reverse: TTAGTTGTGCTTGCCATC |
|  | T-DNA border: TCCAAACTGGAACAACACTCAA |
| Salk_069657 | Forward:ATGATCCTGAGCTTGTTG |
|  | Reverse: CTAATTCCAAGGTTGTGAC |
|  | T-DNA border: TCCAAACTGGAACAACACTCAA |
| Detection of gene expression as shown in Figure 2A | |
| *GPDHc1* | Forward: TCTGGGATCGTTCAATGTCTC |
|  | Reverse: TGCTGGCAAAGGGTGAAATA |
| *GLI1* | Forward: GATTTGGCAGCAATGGTA |
|  | Reverse: TTAGATAGAGAGGTCAGCG |
| *FAD-GPDH* | Forward: ATGTCTCTCGCTTCCATTC |
|  | Reverse: TTAGTTGTGCTTGCCATC |
| *Actin7* | Forward: GCCAAGAGCAGTTCTTCA |
|  | Reverse: CACCACGAACCAGATAAG |
| Specific primers used in real-time reverse transcription PCR | |
| *FAD-GPDH* | Forward: ATTCTAGCAAGTGAACA |
|  | Reverse: GTGTAAGTAAATCTCTGA |
| *CYCB1;1* | Forward: TCCGATTCGAGCTAAACCCGGAAA |
|  | Reverse: CCACCTGTGGTGGCCAAATTTCTT |
| *CDKA;1* | Forward: CGTGGAATTGCGTATTGCCACTCT |
|  | Reverse: AGCAAGCTTCAGTGAGTTTGTGCG |
| *PIN1* | Forward: CTGGTCCCTCATTTCCTTCA |
|  | Reverse: TTTGGCAAACACAAACGGTA |
| *PIN7* | Forward: ATTACGTGGAGACCTATTGCG |
|  | Reverse: TGTACTCAAGATTGCGGGATG |
| *UBQ10* | Forward: GGCCTTGTATAATCCCTGATGAATAAG |
|  | Reverse: AAAGAGATAACAGGAACGGAAACATAGT |
